# Gender gaps in healthy life expectancy as indicators of inequality for disability and chronic disease: cross-sectional evidence from 24 countries, years 2014–2019

**DOI:** 10.1136/bmjopen-2024-096968

**Published:** 2025-11-19

**Authors:** Vanessa Di Lego, Marília R Nepomuceno, Cassio M Turra

**Affiliations:** 1 Demography, Cedeplar, Universidade Federal de Minas Gerais, Belo Horizonte, Brazil; 2 Digital and Computational Demography, Max Planck Institute for Demographic Research, Rostock, Germany

**Keywords:** Aging, Mortality, Health Surveys, Health Equity

## Abstract

**Abstract:**

**Objective:**

Gender gaps in healthy life expectancy are frequently used as indicators of health inequality between women and men. However, total gaps can be misleading—masking critical disparities such as women living longer yet spending more years with disability or illness, or men experiencing premature mortality. We therefore critically evaluate whether these gaps accurately capture gender-based health.

**Design:**

We estimate gender gaps in disability- and chronic disease-free life expectancy using the Sullivan method and decompose those gaps via the continuous-change approach to distinguish mortality from morbidity contributions. Data are drawn from the harmonised Gateway to Global Aging Data and the UN life tables from the 2022 Revision of World Population Prospects for all countries, except England, where the life tables are from the UK Office for National Statistics.

**Setting:**

The analysis is performed on 24 countries and regions, including the USA, England, South Korea, China, India, Mexico and 19 European Union countries for the years 2014–2015 and 2017–2019 (N=201 723).

**Main outcome measures:**

The main outcomes are gender gaps in disability- and chronic disease-free life expectancy and the contribution of mortality and health in explaining the gender gap.

**Results:**

Gender gaps in disability-free life expectancy ranged from −0.37 years (Portugal) to almost 5 years (South Korea), with most European countries showing female advantages of 3.0–3.5 years, while minimal gaps were observed in China, Mexico and India (0.4–0.9 years). Decomposition revealed striking inconsistencies between total gaps and underlying components—South Korea’s 4.9-year gap reflected a survival advantage outweighing disability disadvantage by 13-fold, while Portugal’s −0.37-year gap masked opposing contributions (mortality: +2.3; disability: −2.7). Chronic disease-free life expectancy showed female disadvantage in most countries, especially Portugal (−2.3), Korea (−1.6) and Mexico (−1.9).

**Conclusions:**

Using gender gaps in healthy life expectancy as a metric for gender inequality in health is misleading. Countries with very different levels of development, healthcare systems and gender roles can have similar gender gaps, but substantial differences in the levels of mortality and health. Because these gaps mask important underlying differences in health and mortality between women and men, caution is warranted when using them.

**STRENGTHS AND LIMITATIONS OF THIS STUDY**

- Novel and broad cross-country comparison, using detailed and harmonised health data for 24 countries from three major world regions.
- This study uses the continuous-change approach to decompose total gender gaps in disability- and chronic disease-free life expectancy into contributions of health and mortality.
- Uses a limited number of health variables and years in the analysis, since it depended on harmonised health data available.

## Introduction

 It has been widely documented that women live longer than men, but spend more years in poorer physical, self-rated and cognitive health at older ages.^1^,^6^ They also experience higher morbidity from acute and chronic conditions, and more short-term disability.^7^,^10^ This phenomenon is known in the literature as the male-female health-survival paradox, and researchers often use gender gaps in healthy life expectancy to evaluate health disparities between women and men.^11^,^15^ When it comes to healthy life expectancy, gender gaps are calculated by comparing the number of years that women and men are expected to live in good health.^13 16 17^ This comparison can be expressed as a ratio or an absolute difference. These measures are then used to make inferences about gender discrepancies in health and whether women and men have higher or lower inequality in terms of health and mortality in a population.^2 7 18 19^ Policymakers also rely on such gender gaps to benchmark countries, monitor changes over time and age and assess whether countries are closing or widening gender inequality in health.^20^,^22^ Gaps in healthy life expectancy are also used to construct aggregate indicators of gender inequality. The Global Gender Gap reported by the World Economic Forum,^20^ the WHO European Health Equity Status Report initiative and the Gender Equality Index, are examples of such indicators. They are often used to evaluate gender inequalities in health and mortality^21^ and implement policy action for health equity.^23^

However, it can be misleading to use gender gaps in healthy life expectancy as indicators of gender inequality in health. This is due to the way that gaps in healthy life expectancy are estimated, which incorporates both the proportion of women and men who are unhealthy at a specific age and their mortality rates. The absolute difference in years between their healthy life expectancy is then used as a gender gap in health and mortality. Gaps are often used to measure the difference between two quantities.^24^ These measures are then used to make inferences about gender discrepancies in health.^2 7 18 19^ Indeed, gaps can be robust ways to compare quantities like life expectancy, which depend solely on mortality—a single state that represents the very final stage of the decline of one’s health. However, including other health domains in indicators—as is the case with healthy life expectancy—leads to much more complex interpretations.^25^

While population health and mortality are closely linked, they each exert different impacts on men and women. Hence, it is challenging to interpret gender gaps in healthy life expectancy as unique markers of inequality, as many aspects can be overlooked. While healthy life expectancy remains a valuable health measure, our focus is on examining whether gender gaps in these measures appropriately capture inequality between women and men. Some studies have attempted to distinguish these differences but have primarily focused on countries or regions that share similar gender roles and societal values^26^,^28^ or used health indicators that were less detailed and lacked standardisation, which could have compromised the accuracy of results.^29^

In this paper, we critically assess whether gender gaps in health expectancy are a robust metric for capturing gender inequality in health outcomes in a diverse set of 24 countries from three major world regions, including the USA, England, South Korea, China, India, Mexico and 19 European Union countries.

## Methods

### Data and health variables

Data are from the Gateway to Global Aging, with harmonised versions of the Health and Retirement Study (HRS) sister studies^30^ from the Programme on Global Aging, Health and Policy. The harmonisation procedure follows the RAND HRS conventions of variable naming and data structure, which allows for proper cross-country comparisons. HRS-sister studies use complex survey sample designs based on multi-stage probability samples and sometimes stratified cluster sampling. For more information on the specific sample designs and the construction of sample weights for each survey, refer to online supplemental material under Country-Specific Details. We have used the cross-sectional individual-level respondent weights provided for all harmonised surveys. We used pooled data available for the years 2014–2015 for HRS (USA), ELSA (England), KLoSA (South Korea), CHARLS (China), MHAS (Mexico) and Europe (SHARE), with the exception of India, where LASI was carried out between 2017 and 2019. We selected the periods for which the greatest number of comparable surveys from various countries was available, taking into account all relevant health indicators. We focus on only one period; thus, we use the data as cross-sectional, accounting for respondents’ health during a single period. An overview of the samples is displayed in table 1.

**Table 1:** Overview of countries, waves, covering period and sample size

Country/region	Survey	Waves	Covering period	N=(unweighted)‡
USA	HRS	Wave 12	2014–2015	18 747
Europe	SHARE†	Wave 6	2014–2015	66 708
England	ELSA	Wave 7	2014–2015	8150
China	CHARLS	Wave 3	2014–2015	15 910
India	LASI	Wave 1	2016–2017*	71 863
Korea	KLoSA	Wave 5	2014–2015	7028
Mexico	MHAS	Wave 4	2014–2015	13 317
			N total	201 723

We use the data as cross-sectional only and focus on a single period, which is the year for which there was the largest number of comparable surveys available. All analyses are adjusted considering the weighted sample size and accounting for the complexity of each survey design.

Source: Gateway to Global Aging Data, Produced by the Programme on Global Aging, Health and Policy, University of Southern California, with funding from the National Institute on Aging (R01 AG030153).

* Data for India refers to the year 2016/2017 and not 2014/2015. This was the closest year available to compare with other samples.

† Countries only added in wave 7 and thus not included in this study: Finland, Lithuania, Latvia, Slovakia, Romania, Bulgaria, Malta and Cyprus.

‡ Sample size refers to the total number of observations.

We focus on two health variables: disability and chronic diseases, since they are the harmonised variables that were available for the largest set of diverse countries as possible and are the most used health dimensions in constructing health expectancies.^31 32^ Disability is defined as a survey respondent reporting any difficulty with the 5-item list of activities of daily living (ADLs), which include bathing, dressing, eating, getting in and out of bed and using the toilet. Chronic disease is defined as a respondent reporting being diagnosed with at least one of six doctor-diagnosed conditions present in all harmonised country surveys: arthritis, cancer, diabetes, heart conditions, lung disease and stroke. This set of health variables had very few missing cases; thus, our strategy was to drop the observations from the analyses. Importantly, there was no difference in the pattern of missing cases between women and men, which is crucial for our study, as shown in more detail in online supplemental table S1, which includes the missing cases for every survey and variable used in the analysis, along with some notes specifically related to the survey. Finally, we focus on healthy life expectancy at age 60 because this age marks the onset of more pronounced gender disparities in health outcomes, follows the methodology used by major international reports that monitor gender health inequalities, and corresponds to the age range captured by the health surveys in our analysis. For more details on the health data, refer to online supplemental material section on materials and table S3 for sample characteristics, following the GATHER statement for reporting global health estimates.^33^ For more details on how the harmonisation procedure is implemented across surveys, refer to the full protocol available from the Gateway to Global Aging Data (https://g2aging.org/).

For mortality data, we use publicly available UN life tables from the 2022 Revision of World Population Prospects (United Nations 2022) for all countries. The only exception is England, where the life tables are from the UK Office for National Statistics (ONS), available at https://www.ons.gov.uk/, as the ELSA study does not include Wales.

### Estimating and decomposing gender gaps in healthy life expectancy

We first estimate disability-free life expectancy (DFLE) and chronic-disease-free life expectancy (CFLE) at age 60 using the Sullivan method.^34^,^36^ ‘Unhealthy’ is defined as having reported any limitations in ADL due to disabilities for DFLE or been diagnosed by a physician with at least one chronic disease for CFLE.

Second, we calculate the prevalence of individuals with limitations in disability and of at least one chronic disease for each country by 5-year age groups using the weighted proportions of women and men who reported these conditions in surveys. We then combine the estimated prevalence with country-specific life tables to compute healthy life expectancies. DFLE is defined as the number of years lived free of disability, while CFLE is the number of years lived without chronic diseases. We calculate the gender gap in DFLE as $\Delta_{DFLE}^{\;}={DFLE}^{Women}-{DFLE}^{Men}$and the gender gap in CFLE as $\Delta_{CFLE}={CFLE}^{Women}-{CFLE}^{Men}$.

Third, we decompose the gap into its health and mortality components. Decomposition methods are widely used tools to explain gaps in aggregate indices, such as life expectancies and healthy life expectancies. The goal of decomposition is to attribute the gap in aggregate indices to the contribution of underlying factors. For example, imagine two populations with different life expectancies. To understand why one population lives longer than the other, it is important to know which age groups contribute the most to explain this difference. Decomposition methods allow us to break down the overall gap and determine, for example, how much is due to higher infant mortality or how much is from higher old-age mortality. In other words, decomposition methods help us understand where gaps come from. In this paper, we apply the continuous change decomposition method^37^,^39^ and split the gender differences in DFLE and CFLE at age 60 into mortality and disability/chronic effects (see online supplemental material section on Methods for more details on the Sullivan method and the decomposition approach). This allows us to estimate the contribution of health and mortality in explaining the gap between women and men. The sum of these two components corresponds to the total gender gap. When the total gender gap is positive, it indicates that women have higher healthy life expectancy than men, which is known as the women’s advantage in healthy life expectancy. In such cases, when both components of mortality and health are positive, they both increase the gender gap. On the other hand, when one component is positive and the other is negative, they result in a narrower gap.

The sum of these two components corresponds to the total gender gap. When the total gender gap is positive, it indicates that women have a higher healthy life expectancy than men, which is known as women’s advantage in healthy life expectancy. In such cases, when both the components of mortality and health are positive, they tend to increase the gender gap. On the other hand, when one component is positive and the other is negative, they can lead to a narrower gap.

### Patient and public involvement

No patients or members of the public were involved in the study.

## Results

### Age-specific prevalence of unhealthy individuals

**Figure 1:**
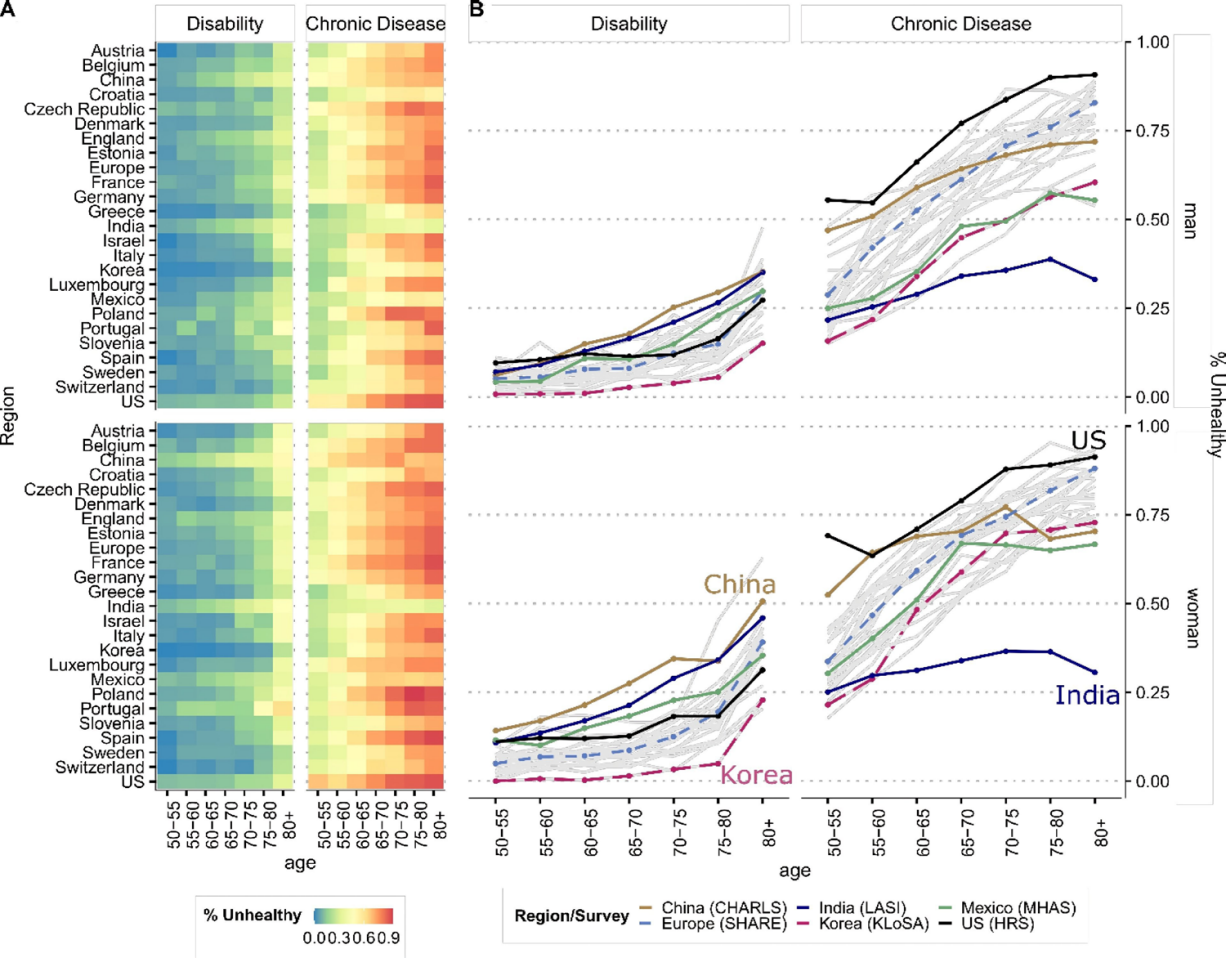
Prevalence of unhealthy women and men by disability and physician-diagnosed chronic disease by age. All countries are presented in panels A and B. Panel B highlights the survey regions and the age pattern. Disability is based on the 5-item list of activities of daily living, which include bathing, dressing, eating, getting in and out of bed and using the toilet. Chronic disease is defined as having reported a diagnosis by a doctor with at least one of six doctor-diagnosed conditions present at all country surveys that were harmonised: (1) arthritis, (2) cancer, (3) diabetes, (4) heart conditions, (5) lung disease, (6) stroke. For reference, see online supplemental material section on Materials for more details on how diagnoses are defined and which criteria are used. For country and region-specific profiles for each condition, see online supplemental figures S1–S8 in the SI. Source: Gateway to Global Aging Data, Produced by the Programme on Global Aging, Health and Policy, University of Southern California with funding from the National Institute on Aging (R01 AG030153).

Figure 1 shows that the prevalence of being diagnosed with at least one chronic disease is higher than that of a reported disability (panel A). Most countries have a strong age gradient in health for both sexes (panel B, see online supplemental figures S1–S8 in the SI for all countries and separately for each chronic disease and disability). Within countries, women experience a faster rate of increase in disability with age, resulting in a greater health burden for women at younger ages relative to men (ranging from 14% to 24.2% in women across countries, while from 4% to 16% in men, p<0.001, see online supplemental table S6). Chinese and Indian women not only experience the highest rates of disability overall (24.2% and 19.4%, respectively, compared with 16.1% and 13.8% of their male counterparts, p<0.001), but their disability prevalence at ages 60–65 is only observable among men at ages 70–75, almost a 10-year difference.

Additionally, panel B in figure 1 shows that the USA has the highest prevalence of at least one chronic disease among women (77.3%) and men (71.9%) of most ages. China follows the USA with a higher prevalence among those under 65, but it levels off as people age. In contrast, other countries have lower prevalence levels, but they grow faster with age. India has the lowest prevalence for both women (30.7%) and men (28.6%).

### Gender gap in healthy life expectancy and the role of health and mortality components

**Figure 2:**
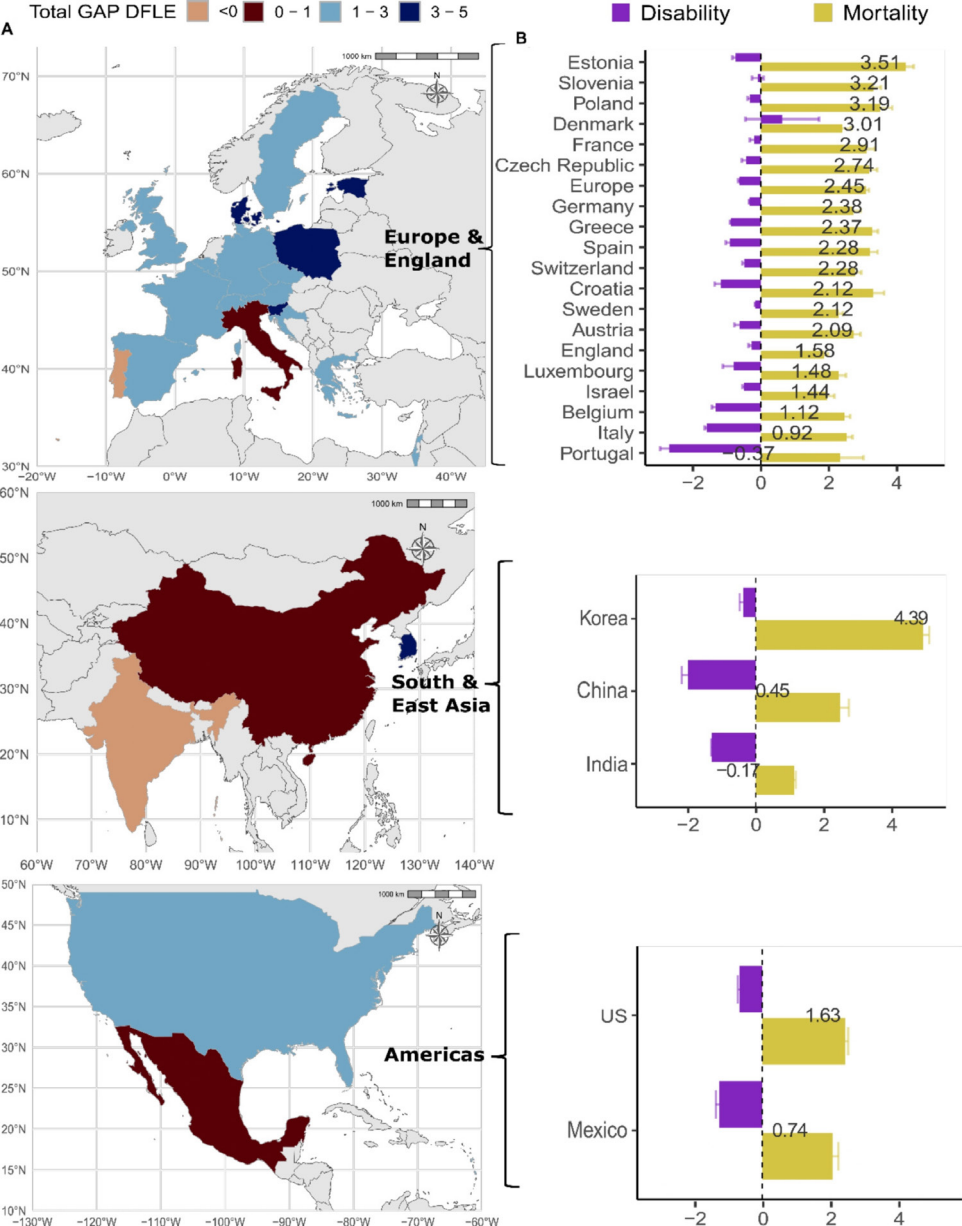
Country-specific gender gap (women−men) in disability-free life expectancy at age 60 (in years) and the contribution of mortality and disability to the total gap for each country. The values for the total gender gap in disability-free life expectancy (DFLE) shown in panel B on the bars are centred on the value. Panel A presents the total DFLE gaps in years and panel B presents the decomposition into mortality and disability effects. Korea refers to South Korea. Panel B ranks the countries from greatest to smallest women’s advantage in the total gap in DFLE in each broad region (Europe and England, South and East Asia, and Americas). Gender gaps are estimated within countries. Disability is defined as a respondent reporting any difficulty with the 5-item list of activities of daily living, which include bathing, dressing, eating, getting in and out of bed and using the toilet. Source: Gateway to Global Aging Data, Produced by the Programme on Global Aging, Health and Policy, University of Southern California, with funding from the National Institute on Aging (R01 AG030153).

Figure 2 shows the total gender gap in disability-free life expectancy (DFLE) by country and world region (panel A) and the contribution of the mortality and disability in explaining this gap at age 60 (panel B) (see online supplemental tables S2 and S3 for the total gender gap and online supplemental tables S4 and S5 for all values for each country with CIs). Different from figure 1, where the prevalence of unhealthy is shown, the total gender gaps in healthy life expectancy are based on the number of years that women and men can expect to live in a healthy state.

Panel A of figure 2 shows the total variation in the gender gap across and within each region. Across all countries considered, women have the highest advantage over men in South Korea, with a difference of almost 5 years. This is followed by countries mainly in Europe and England region, where women have a higher advantage in Estonia (3.5 years), Slovenia (3.2 years), Poland (3.2 years) and Denmark (3 years). In China, Mexico and Italy, women have very little advantage over men (0.4 years, 0.5 years and 0.9 years, respectively). Only in Portugal (−0.37 years) and India (−0.17 years) are women disadvantaged compared with men. The total gap in panel A is explained by the disability and mortality components shown in panel B. The wider gender gap in South Korea is due to women’s remarkable survival advantage, which is 13 times higher than their disability disadvantage relative to men. In Portugal, the total gap is small and negative. The contributions of both disability and mortality are high but act in opposite directions (mortality contribution=2.3 years and disability contribution=−2.7 years, see online supplemental figure S9 for the contribution to the total gender gap for all countries).

**Figure 3:**
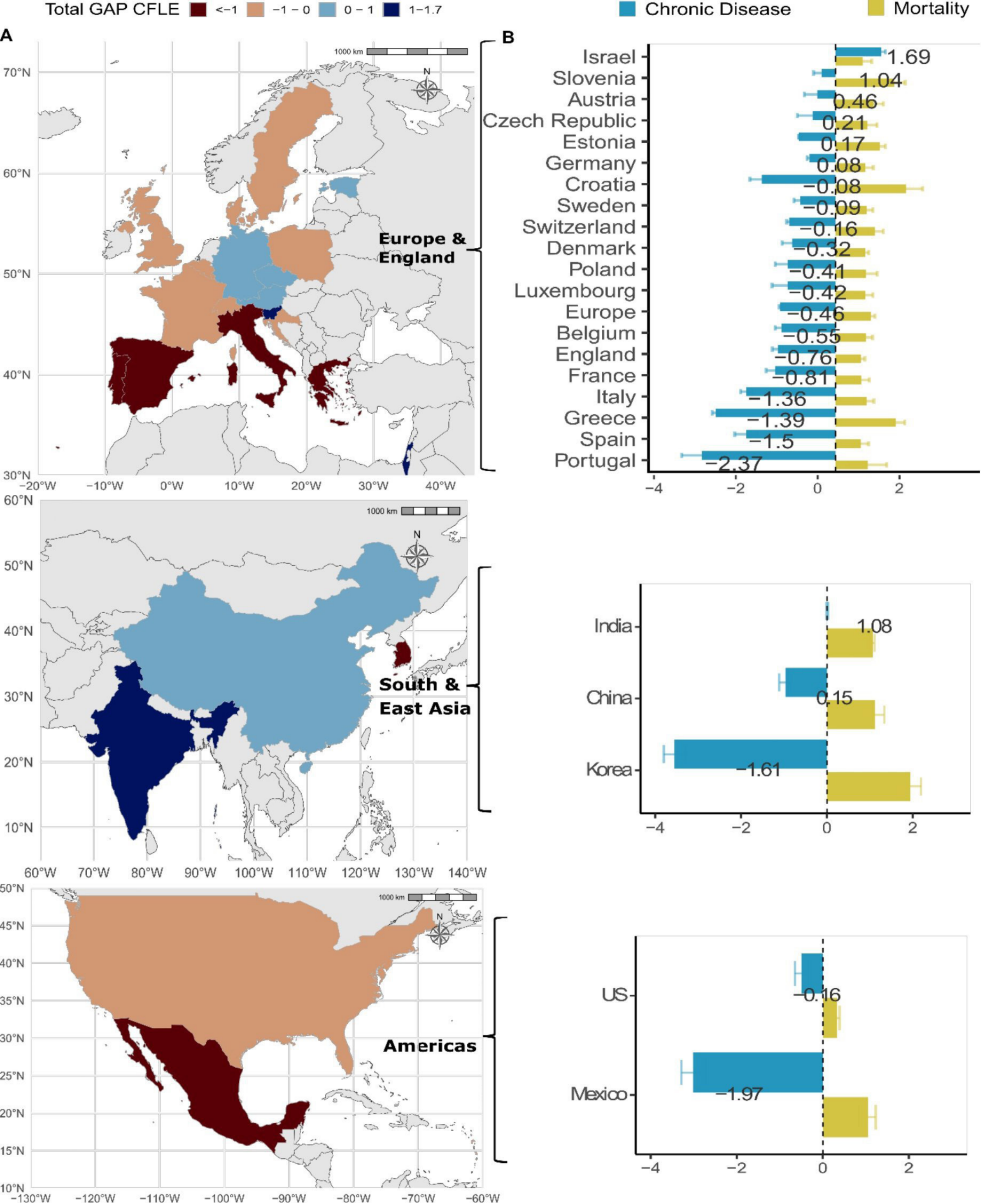
Country-specific gender gap (women−men) in chronic disease-free life expectancy at age 60 (in years) and the contribution of mortality and chronic disease to the total gap by country and region. The values for the total gender gap (women-men) in chronic-disease-free life expectancy (CFLE) shown in panel B on the bars are centred on the value. Korea refers to South Korea. Panel A presents the total CFLE gaps in years, and panel B presents the decomposition into mortality and chronic disease effects. Gender gaps are estimated within countries. Chronic disease is defined as a respondent reporting being diagnosed with at least one of six physician-diagnosed conditions present in all harmonised country surveys: (1) arthritis, (2) cancer, (3) diabetes, (4) heart conditions, (5) lung disease, (6) stroke. Source: Gateway to Global Aging Data, Produced by the Programme on Global Aging, Health and Policy, University of Southern California, with funding from the National Institute on Aging (R01 AG030153).

Figures2  3 highlight different patterns in the gender gap across two very different health indicators, CFLE and DFLE. Figure 3 shows the gender gap in chronic disease-free life expectancy (CFLE), where women are at a disadvantage compared with men in most countries (see online supplemental figures S1–S8 for the contribution of each chronic disease). This disparity is more pronounced in Portugal, South Korea and Mexico, with women experiencing fewer years of life free from chronic diseases than men by 2.3, 1.6 and 1.9 years, respectively. This contrast in the gender gap between CFLE and DFLE suggests that while women might have a comparable or even longer overall life expectancy than men in different contexts, the quality of those additional years may be compromised by the prevalence of chronic diseases.

Most importantly, the analysis of CFLE reveals that countries with similar gaps in chronic disease-free life expectancy do not necessarily share the same profiles in terms of chronic disease and mortality contributions. For instance, despite both Switzerland and the USA having a CFLE gap of −0.16 years, the contributions of mortality and chronic diseases to this gap vary significantly between the two countries. In Switzerland, the impact of mortality and chronic diseases on the CFLE gap is two to three times higher than in the USA.

**Figure 4:**
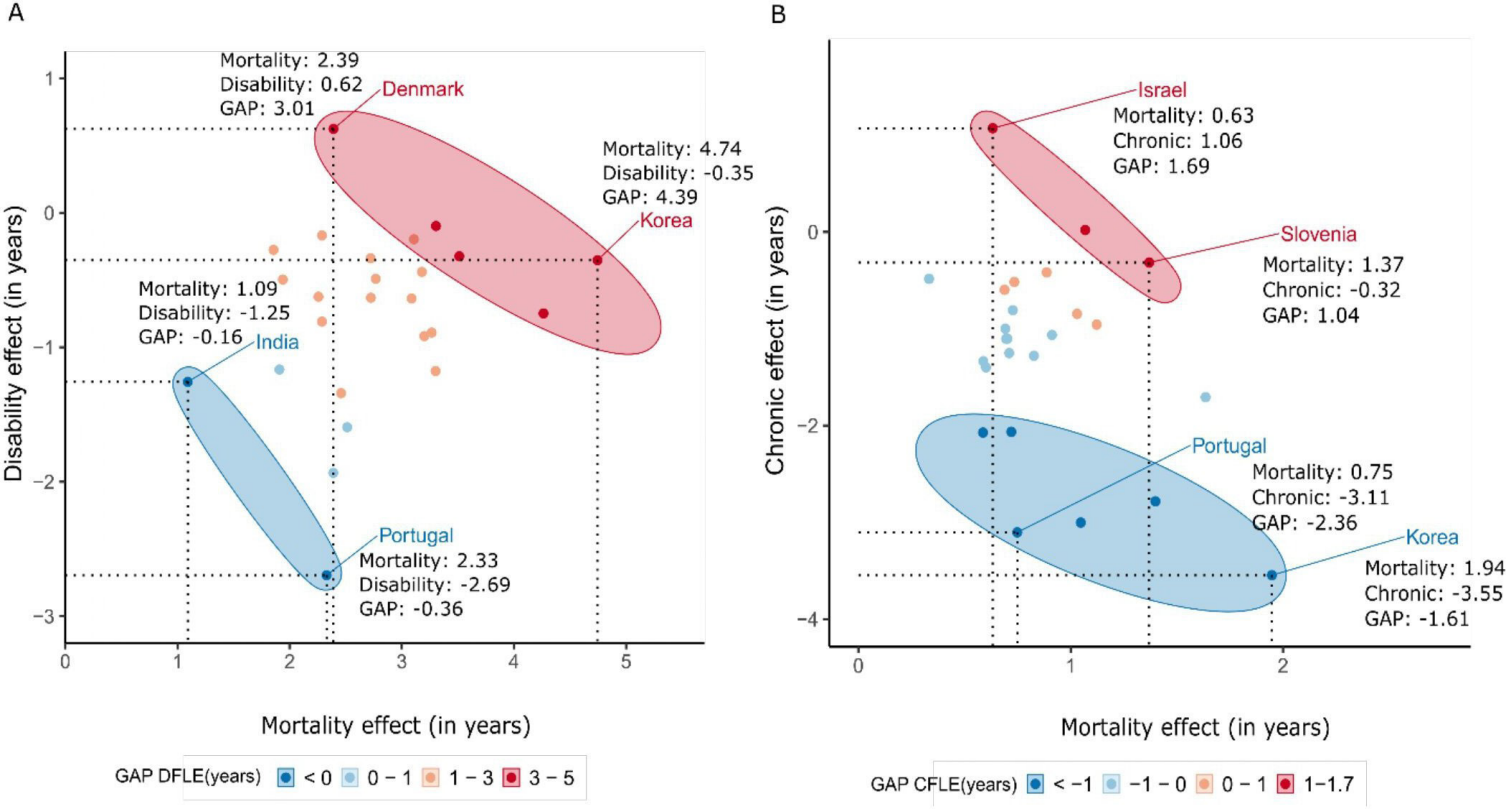
Decomposition of the gender gap (women−men) in disability-free life expectancy (DFLE) at ages 60 into mortality and disability effects (panel A) and in chronic disease-free life expectancy (CFLE) at age 60 into mortality and chronic effects (panel B) by country. Panel A presents selected countries, grouped by their GAP in DFLE (women−men) and the contributions of disability and mortality to the total GAP. Korea refers to South Korea. Panel B presents selected countries, grouped by their GAP in CFLE (women−men) and the contributions of chronic and mortality to the total GAP. Source: Gateway to Global Aging Data, Produced by the Programme on Global Aging, Health and Policy, University of Southern California, with funding from the National Institute on Aging (R01 AG030153).

Finally, figure 4 groups countries according to the overall gender gap in healthy life expectancy and disentangles the mortality, disability and chronic disease components in the gender gaps in DFLE (panel A) and CFLE (panel B) across different countries. It is clear how focusing only on gender gaps in healthy life expectancy can mask significant underlying disparities in health and mortality. For instance, panel A of figure 4 shows how completely disparate countries like India and Portugal are grouped together, with both countries having relatively low gender gap values in DFLE at age 60 (−0.16 for India and −0.36 for Portugal). However, the underlying factors contributing to these gaps reveal a complex interplay between disability and mortality components. The disability component has a significant negative impact on the DFLE gender gap, with −1.25 years for India and −2.69 years for Portugal, indicating that women in these countries have fewer years of disability-free life compared with men. Conversely, the mortality component, which adds 1.09 years in India and 2.33 years in Portugal, works in the opposite direction but does not fully compensate for the negative impact of disability. Likewise, South Korea and Denmark are grouped together among the countries with the widest gender gaps in DFLE, 4.39 and 3.01 years, respectively. However, the gap in South Korea is primarily attributed to the survival advantage that women have over men (4.74 years), which is only slightly offset by a negative contribution from disability (−0.35 years), while in Denmark, the longer life expectancy of women not only stems from a mortality advantage but also from a positive contribution due to disability.

Similar disparities are observed for CFLE, as shown in panel B. Portugal and Korea are now grouped together as countries that exhibit the largest negative gaps, while Israel and Slovenia are grouped as the ones with the largest positive gaps. However, the advantage women hold in CFLE in Israel stems from both mortality and chronic disease contributions being small and positive, while in Slovenia the contribution of chronic disease is negative. The contrasting patterns between disability-free and chronic disease-free life expectancy demonstrate how relying only on gap indicators can mask important underlying health differences.

## Discussion

Gender gaps in healthy life expectancy are often used to measure the level of gender inequality in health across different countries. However, we found little internal consistency between gaps in healthy life expectancy and the health and mortality components contributing to them. These inconsistencies challenge the use of the gender gap based on healthy life expectancy as an appropriate inequality measure, particularly in the context of comparative analyses. Our findings show that the issue with misinterpreting gender gaps as accurate inequality measures holds regardless of which health domain is investigated or how health is defined. Countries with different epidemiological and cultural contexts can have similar gender gaps at a given time, but that does not mean they have the same levels of health inequality. For example, both Portugal and India have similar small and negative gender gaps in DFLE (ie, women have lower DFLE than men), but very different contributions from mortality and health. Likewise, while both Switzerland and the USA have a CFLE gap of −0.16 years, the impact of mortality and chronic diseases on the CFLE gap in Switzerland is two to three times higher than in the USA.

Additionally, when we group countries based on their gender gap, we find that countries from very different regions of the world with varying levels of development, healthcare systems and gender roles can be in the same category. These issues raise questions about whether it is appropriate to rank countries based on gender gaps in healthy life expectancy and whether they are in fact discerning inequalities in health. This is key, as policies aimed at promoting gender equality in health across different countries are already surprisingly poorly designed and implemented, primarily due to a scarcity of relevant data and accurate indicators.^40^

This study makes an important contribution by including a comprehensive comparative analysis that extends beyond Western countries. Previous research has given little attention to countries like China, India and South Korea, and even fewer studies have focused on Latin American countries such as Mexico and other middle- and low-income nations.^1041^,^44^ Global comparisons in health studies often lack detailed, harmonised health indicators.^29^ This is especially important when examining patterns by gender, as development levels and societal roles of men and women in different countries may directly or indirectly impact health and mortality indicators.^45^,^48^

The main reason why the gender gap in healthy life expectancy does not accurately capture inequality is due to the very particular documented relationship between health and mortality and the specific role of certain health conditions for both genders. Despite living longer than men, women experience poorer health for most outcomes,^29 11^,^13 15 49^ facing a higher burden of non-lethal, debilitating chronic conditions, such as arthritis,^49 50^ while men experience higher levels of diabetes and heart disease, conditions that are linked to higher mortality risk.^51^ Therefore, gender differences in healthy life expectancy make it difficult to disentangle the contributions of health and mortality. In principle, HLE gender gaps can be informative markers of inequality—but only when disaggregated by age, type of disability and social context. Other alternative indicators include: (1) severity-adjusted life expectancy distinguishing mild versus severe disabilities; (2) cause-specific mortality ratios by gender across different health conditions; (3) gender gaps in healthcare utilisation and access; (4) preventable mortality ratios by gender; (5) health system responsiveness measures by gender.

It is important to acknowledge that this study has some limitations. Despite efforts to harmonise the variables, disease diagnosis is performed differently across countries. In some settings, the low prevalence of chronic diagnosed diseases may reflect the low quality of healthcare, such as in the case of India.^52^,^54^ ^55^ Another relevant difference across countries is who can make the diagnosis. The HRS (USA) study, for example, specifically excludes diagnoses made by nurses/nurse practitioners, chiropractors and dentists, while both CHARLS (China) and LASI (India) allow diagnoses by nurses, practitioners of traditional medicine and other healthcare professionals. It is unclear whether these differences impact diagnoses for each gender in a similar way. However, the objective of this study is not to investigate the factors that determine health status in certain regions. Instead, the focus is on how differences between genders in terms of healthy life expectancy incorporate various elements of health and mortality that raise doubts about its suitability as a measure of gender inequality in health.

Also, our analyses do not use longitudinal data, only cross-sectional data for each country and for a limited period. Considering that health and mortality reflect transitions throughout the life course, it is possible that the assumption of a hypothetical cohort, inherent in cross-sectional data, may affect genders and countries in different ways, potentially biasing our comparisons.^56^ On the other hand, the use of cross-sectional data broadens the possibilities for comparison across a larger number of countries and has been used in the absence of longitudinal data.

## Conclusion

Closing the gender gap in healthy life expectancy does not necessarily mean reducing health inequality between women and men. Therefore, we recommend caution when using gaps in summary indicators like health expectancy to measure gender inequalities in health and suggest using separate indicators for health and mortality. We also call for the development of new summary measures that accurately reflect gender inequalities in health across countries.

## Supplementary material

10.1136/bmjopen-2024-096968 online supplemental file 1

## Data Availability

Data may be obtained from a third party and are not publicly available.
