# Supplementary material for: Gender gaps in healthy life expectancy as indicators of inequality for disability and chronic disease: cross-sectional evidence from 24 countries, years 2014–2019
Source: BMJ Open. 2025 Nov 19;15(11):e096968. doi: 10.1136/bmjopen-2024-096968 (PMC12636968; doi:10.1136/bmjopen-2024-096968)
Supplement: online supplemental file 1 [file bmjopen-15-11-s001.docx]

**Supporting Information for**

Gender Gaps in Healthy Life Expectancy as Indicators of Inequality for Disability and Chronic Disease: Evidence from 24 Countries, years 2014–2019

Vanessa Di Lego^1,2^*, Marília R. Nepomuceno^3^, Cássio M. Turra ^1,4^

^1^ Department of Demography, Faculty of Economics, Federal University of Minas Gerais (UFMG), Belo Horizonte, Brazil – affiliation changed upon submission

^2^ Wittgenstein Centre for Demography and Global Human Capital (IIASA, OeAW, Univ. Vienna), Vienna Institute of Demography at the Austrian Academy of Sciences – affiliation where most of the work was conducted

^3^ Max Planck Institute for Demographic Research, Rostock, Germany.

^4^ Department of Demography, Faculty of Economics, Cedeplar, Federal University of Minas Gerais (UFMG), Belo Horizonte, Brazil

*Corresponding author

**Email:**  vdilego@cedeplar.ufmg.br

**This file includes:**

Methods

Materials

Figures S1 to S9

Tables S1 to S6

SI References

Methods

1. Computing Disability- and Chronic-free life expectancy

We first estimate the disability-free life expectancy (*DFLE*) and the chronic-free life expectancy (*CFLE*) for ages 60 y and over using the Sullivan Method ^1,2^.

The number of person-years lived free of disability (${{{}_{n}L}_{x}^{i}}$) is calculated as,

$\begin{aligned} {{{}_{n}{{L_{x}}}}^{i}}={}_{n}{{L_{x}}} \left( 1- {{{}_{n}\pi}_{x}} \right) \#(1) \end{aligned}$

Where ${{}_{n}{{L_{x}}}}^{i}$ is the number of person-years lived without disability between ages $x$ and $x+n$, ${}_{n}{{L_{x}}}$ is the total number of person-years lived in the age group $x$ and $x+n$, and ${{}_{n}\pi}_{x}$ is the proportion of disabled individuals in the age group $x$ and $x+n$. Then, life expectancy free of disability (*DFLE*) is calculated as:

$$\begin{aligned} {DFLE}_{x}=\frac{\sum_{k=x}^{w} \left( {{}_{n}{{L_{k}}}}^{i} \right)}{l_{x}} \#(2) \end{aligned}$$

The same is for chronic-free person-years lived, however with the prevalence for at least one chronic condition instead of prevalence of ADLs, and we call the person-years derived by the same process as ${{}_{n}L}_{x}^{c}$. Hence, life expectancy free of chronic disease (*CFLE*) is:

$$\begin{aligned} {CFLE}_{x}=\frac{\sum_{k=x}^{w} \left( {{}_{n}{{L_{k}}}}^{c} \right)}{l_{x}} \#(3) \end{aligned}$$

The 95% confidence intervals for the *DFLE* and *CFLE* are computed directly from the standard errors derived from the weighted prevalence from each survey. For more details on how the weights are constructed for each survey, refer to data documentation on <https://g2aging.org/survey-overview>.

1. Gender Gap

The total gender gap in *DFLE* is calculated as:

$$\begin{aligned} \Delta_{xDFLE}={DFLE}_{x}^{Women}-{DFLE}_{x}^{Men} \#(4) \end{aligned}$$

And the gender gap in *CFLE* as:

$$\begin{aligned} \Delta_{xCFLE}={CFLE}_{x}^{Women}-{CFLE}_{x}^{Men} \#(5) \end{aligned}$$

In this way, larger gaps imply more advantage in favor of women and smaller gaps less advantage. Negative gaps imply that men have more advantage than women. Gaps are in absolute years.

1. Decomposing the gender gap

We used a decomposition method to split the gender differences in $\Delta_{xDFLE}$ and $\Delta_{xCFLE}$ at ages 60 years and over into mortality and disability/chronic effects by five-year age groups. Decomposition methods are widely used tools to explain gaps in aggregate indices, such as life expectancies and healthy life expectancies. The goal of decomposition is to attribute the gap in aggregate indices into the contribution in the underlying factors. Imagine two populations with different life expectancies. To understand why one population lives longer than the other, it is important to know which age groups contribute the most to explain this difference. Decomposition methods allow us to break down the overall gap and determine, for example, how much is due to higher infant mortality, how much is from higher old-age mortality, and so on. In other words, instead of just knowing that there's a gap, decomposition methods help us to understand where the difference comes from. There are many different decomposition methods available. In this study, we apply the Continuous Change Decomposition Method. To decompose the gap, we apply the continuous change decomposition method, which assumes that covariates change continuously along an actual or hypothetical dimension, such as between two periods or between two populations ^3–5^. Such changes can be approximated by a linear combination of *n* partial derivatives of the function with respect to the covariates ^3^. Numerical integration is used to obtain the total contribution of the covariates for the variation of the aggregate measure. This method is very flexible, and can be used for decomposing gaps in different aggregate measures. For more details, refer to ^5^. Previous research has employed the methodology to estimate gaps in disability for Latin American and Caribbean (LAC) countries ^6^. By applying this method, we assume that changes in mortality and health occur gradually between the female and male populations 3–5. As van Raalte and Nepomuceno (2020) explain: “If we could split the time or space interval into many, many smaller intervals, then we could estimate the changes needed by the covariates (for instance age-specific mortality) to change the aggregate function (for instance life expectancy) from one population to the next”. In this study, each tiny change in age-specific mortality and health measures in the healthy life expectancy can be approximated by a linear combination of n partial derivatives of the function to calculate healthy life expectancy with respect to its covariates: age-specific mortality and health measures. These can then be aggregated, using numerical integration to give the total contribution of the covariates to the change in the aggregate measure.”. This method is very flexible, and can be used for decomposing gaps in different aggregate measures. For more details, refer to 5. Previous research has employed the methodology to break down gender gaps in disability for Latin American and Caribbean (LAC) countries 6.

Materials

1. **Overall Health Data**

Health data for all countries is from the Gateway of Global Ageing, including doctor diagnosed chronic conditions and disability. We follow the recommendation by the Gateway of Global Ageing official report that for harmonization purposes, general diagnosis of chronic medical conditions (e.g. heart diseases), rather than a more specific condition (e.g. congestive heart failure), may be used to maximize the number of studies that may be included ^7^. We thus use the diagnosed chronic conditions surveyed for all HRS-sister studies and the following **disease variables:** RwDIABE, RwCANCRE, RwLUNGE, RwHEARTE, RwSTROKE, and RwARTHRE. There are indicator variables denoting whether or not the Respondent reports a doctor has ever told her/him that s/he had the specified condition. The conditions are 1) RwDIABE. diabetes or high blood sugar; 2) RwCANCRE. cancer or a malignant tumor of any kind except skin cancer; 3) RwLUNGE. chronic lung disease except asthma such as chronic bronchitis or emphysema; 4) RwHEARTE. heart problems, which include heart attack, coronary heart disease, angina, congestive heart failure, or other heart problems; 5) RwSTROKE. stroke or transient ischemic attack (TIA); and 6) RwARTHRE. arthritis or rheumatism.

Specifically for the US, the Rand HRS 1992_2018v1 files we use as basis for these doctor-diagnosed conditions, with the exception of cases that dispute a report from a prior wave, each of these variables is set to "yes" if the Respondent answered yes to the pertinent question in the current or any prior wave, and to "no" if the Respondent responded no at the current and all prior waves. We have constructed three new variables that capture prevalence of chronic conditions. “chronic” refers to having at least one of the conditions cited above. “chronic_sum” refers to the total number of conditions ever diagnosed. “chronic_severe” is a dummy variable that measures comorbidity, where an individual is diagnosed with having three or more of those conditions. **Robustness Checks.** At first, in order to evaluate further differences in onset of disease, we included the variable RADIAGDIAB, which indicates the age at which the respondent was first diagnosed with diabetes. RwRECCANCR indicates the most recent age at which the respondent was diagnosed with cancer. Respondents are asked the year in which they were most recently diagnosed with cancer, and these responses are converted to their age at diagnosis. Previous responses are carried forward if the respondent does not report a new cancer diagnosis. RwRECHRTATT indicates the most recent age at which the respondent had a heart attack. RAFRHRTATT indicates the age at which the respondent had their first heart attack. However, a first analysis showed that in the sample of HRS and other countries, more than 75% was missing for age variables. So, we did not use these variables in the analysis and caution researchers when choosing to work with these variables. However, for our other variables of interest among the eligible respondents, the percentage of missing values was extremely low, and with no differential pattern by gender for most surveys, so these cases were simply dropped. Table S1 presents the missing values for all surveys and for each health variable used. Mexico and China have special cases that are explained in the notes and also present in their respective data documentation. Again, there is no specific gender pattern in the missing values, so we dropped these cases from the initial sample.

Table S1: %Distribution of missing values by survey, gender, and health variables of interest

| **Variables** | **USA (HRS) WAVE 12** | | **India (LASI) WAVE 1** | | **Korea (KLoSA) WAVE 5** | | **England (ELSA) WAVE 7** | | **Europe (SHARE) WAVE 6** | | **Mexico (MHAS) WAVE 4*** | | **China (CHARLS) WAVE 3**** | |  |
| --- | --- | --- | --- | --- | --- | --- | --- | --- | --- | --- | --- | --- | --- | --- | --- |
|  | W | M | W | M | W | M | W | M | W | M | W | M | W | M |  |
|  | % | | | | | | | | | | | | | |  |
| Cancer | 0.29 | 0.26 | 0.21 | 0.35 | 0.00 | 0.00 | 0.00 | 0.00 | 0.21 | 0.20 | 6.66 | 8.83 | 16.63 | 17.37 |  |
| Heart Disease | 0.28 | 0.22 | 0.21 | 0.35 | 0.00 | 0.00 | 0.00 | 0.00 | 0.19 | 0.17 | 6.78 | 8.86 | 16.86 | 17.52 |  |
| Diabetes | 0.22 | 0.18 | 0.21 | 0.37 | 0.00 | 0.00 | 0.00 | 0.00 | 0.20 | 0.19 | 4.97 | 7.09 | 17.33 | 17.92 |  |
| Arthritis | 0.18 | 0.18 | 0.29 | 0.43 | 0.00 | 0.00 | 0.00 | 0.00 | 0.16 | 0.18 | 4.39 | 7.14 | 16.64 | 16.90 |  |
| Lung Disease | 0.14 | 0.16 | 0.21 | 0.35 | 0.00 | 0.00 | 0.00 | 0.00 | 0.21 | 0.20 | 6.21 | 8.35 | 16.55 | 17.18 |  |
| Stroke | 0.14 | 0.13 | 0.21 | 0.35 | 0.02 | 0.00 | 0.00 | 0.00 | 0.21 | 0.20 | 6.31 | 8.17 | 16.28 | 17.12 |  |
| ADLs | 0.10 | 0.06 | 0.36 | 0.54 | 0.00 | 0.00 | 0.00 | 0.06 | 0.22 | 0.20 | 12.41 | 15.05 | 3.85 | 3.52 |  |
| hhidpn | 0.00 | 0.00 | 0.00 | 0.00 | 0.00 | 0.00 | 0.00 | 0.00 | 0.00 | 0.00 | 0.00 | 0.00 | 0.00 | 0.00 |  |
| Wave | 0.00 | 0.00 | 0.00 | 0.00 | 0.00 | 0.00 | 0.00 | 0.00 | 0.00 | 0.00 | 0.00 | 0.00 | 0.00 | 0.00 |  |
| Year | 0.00 | 0.00 | 0.00 | 0.00 | 0.00 | 0.00 | 0.00 | 0.00 | 0.00 | 0.00 | 0.00 | 0.00 | 0.00 | 0.00 |  |
| raestrat | 0.00 | 0.00 | 0.00 | 0.00 | 0.00 | 0.00 | 0.00 | 0.00 | 0.00 | 0.00 | 0.00 | 0.00 | 0.00 | 0.00 |  |
| raehsamp | 0.00 | 0.00 | 0.00 | 0.00 | 0.00 | 0.00 | 0.00 | 0.00 | 0.00 | 0.00 | 0.00 | 0.00 | 0.00 | 0.00 |  |
| rwtresp | 0.00 | 0.00 | 0.00 | 0.00 | 0.00 | 0.00 | 0.00 | 0.00 | 0.00 | 0.00 | 0.00 | 0.00 | 0.00 | 0.00 |  |
| Age | 0.00 | 0.00 | 0.00 | 0.00 | 0.00 | 0.00 | 0.00 | 0.00 | 0.00 | 0.00 | 6.79 | 8.84 | 0.49 | 0.36 |  |
| Notes: *Wave 4 of MHAS the study protocol included a follow-up interview with all the surviving respondents that had completed at least one interview since 2001. In addition, the protocol included those from the new sample added in 2012 that could not be contacted in 2012, contributing to some missings. See Wong et al.., (2017) for more details. ** In Wave 3 of CHARLS respondents have missing values due to an error in the Life History Survey code for "ever had" responses for doctor diagnosed health problems. These cases were excluded from the analysis. | | | | | | | | | | | | | | |  |
|  |  |  |  |  |  |  |  |  |  |  |  |  |  |  |  |
|  |  |  |  |  |  |  |  |  |  |  |  |  |  |  |  |
|  |  |  |  |  |  |  |  |  |  |  |  |  |  |  |  |

**Country specific details. US (HRS).** We are using the Harmonized version B HRS: 37,495 observations. October 2018- There is a new updated version C, until 2019 that was updated now in 2022 and contains 42,233 observations. It is a Respondent level file so each row represents a unique Respondent. This leaves us with 18,747 observations using only wave 12 (year 2014) of HRS. Since the HRS sample is a multi-stage probability sample of the United States, with oversamples of Blacks and Hispanics, unbiased estimates of population parameters require the use of these sampling weights. The HRS provides different types of weights, depending on the research unit analysis: household or respondent level. They are both constructed using the poststratification method. At the household level, population totals of household cohorts, the race/ethnicity of household members, coupleness status, household type, and counts of financial units are used before 2016. In 2016, household poststratification additionally controls for region, education of household members, and labor force status of household members for the LBB cohort households. Respondent-level poststratification differs by dwelling type. Community respondents are post-stratified to population totals of cohort, marital status, sex, and race/ethnicity. Because we focus on individual level, we use the individual level respondent weights to account for non-response adjustment (variable “rwtresp”), and strata and sample weights to account for complex survey design (variables "raestrat" and "raehsamp"). For more details on how the weights are constructed, refer to <https://hrsdata.isr.umich.edu/sites/default/files/documentation/data-descriptions/trk2018v2a.pdf>.

**Mexico (MHAS).** Version B.4 incorporates the latest released version of MHAS data, and adds several new variables. It contains 22,016 observations or rows- 22016. We are using the Harmonized VERSION B.4 (2001-2015), February 2022, for the MHAS data. The Mexican Health and Aging Study (MHAS) is a longitudinal household survey dataset for the study of health, economic position, and quality of life among the elderly. MHAS datasets as of September 2020. The MHAS (Mexican Health and Aging Study) Version B.4 incorporates the latest released version of MHAS data, and adds several new variables. It contains 22,016 observations or rows. It is a Respondent-level file so each row represents a unique Respondent. We will focus on Wave 4, which is for years 2014/2015. After taking out the non-eligible individuals we have 13,317 observations. For performing all the analyses, person-level weights (RwWTRESP) are used. The person-level weights are taken directly from individual weights, FACTORI, in the MHAS Follow-up Master File, which are constructed to represent the population aged 50 or older. The MHAS person-level weights are based on the birth cohort, household composition, and the place of residence (in urban and rural areas) and geographic areas. Because we focus on the individual level, we use the person-level respondent weights to account for non-response adjustment. For more details on how the weights are constructed, refer to the MHAS website at http://www.mhasweb.org/.

**England (ELSA).** We are using the Version G.2 (2002-2019), July 2021 for The English Longitudinal Study on Ageing (ELSA). It is a longitudinal household survey dataset for the study of health, economic position, and quality of life among the elderly (panel survey of people aged 50 and over and their partners, living in private households in England). Version G.2 incorporates the latest released version of ELSA data, which includes eleven main modules and the associated datasets, and adds variables and observations from Wave 9 with a total of 19,802 observations. It also adds new variables and makes adjustments and corrections. We will focus on Wave 7, nonetheless. The samples have been drawn from households which previously responded to the Health Survey for England (HSE). The seventh wave was conducted between June 2014 and May 2015 and included a refreshment sample selected from HSE 2011-2012. After selecting only the eligible observations, we have a sample size of 8,150. ELSA provides stratification and clustering variables to account for the impact of complex sample design on standard errors. RwSTRAT and RwCLUST have values taken directly from these variables provided by ELSA. In addition, ELSA provides a person-level cross-sectional weight and longitudinal weight. Because we use data as cross-sectional, we use this weight. RwCWTRESP is the person-level cross-sectional weight as defined by ELSA. RwCWTRESP is provided to account for any bias from non-response in order to make the respondent sample more representative of the population. These cross-sectional weights also allow for the inclusion of new respondents from the refreshment samples who would not have a longitudinal weight. Additional information about ELSA can be obtained from ELSA’s website at https://www.elsa-project.ac.uk.

**India (LASI).** The Longitudinal Aging Study in India (LASI) is a multidisciplinary, internationally harmonized panel study designed to be nationally representative of India’s population aged 45 and older. LASI is a joint project of three partnering institutions: International Institute for Population Sciences (IIPS), Harvard T.H. Chan School of Public Health (HSPH), and University of Southern California (USC). The first wave was conducted between 2017 and 2019 in 35 of India’s 36 states and union territories (except Sikkim). This initial sample, as released by USC, included 42,951 households and 72,262 individuals. The LASI sampling plan is complex and was based on the 2011 Indian Census with a multistage, stratified cluster sample design. The sample design includes three distinct selection stages in rural areas and four stages in urban areas. We use Version A.2 that makes corrections using the January 2021 released version of Wave 1 of the LASI data. Eligible cases are 71,863 observations. Harmonized LASI weights are provided at the individual and at the household level. They are constructed in two steps. In a first step, a design weight is created to account for unequal selection probabilities of households, and therefore, individuals within selected households. In a second step, post-stratification weights are generated to correct for differential non-response rates and to bring the sample in line with the reference population as far as the distribution of key socio-demographic variables is concerned. Because we focus on individual level, we use the individual level person respondent weights to account for non-response adjustment (variable “rwtresp”). Additional information about the LASI can be obtained from the LASI website at https://lasi-india.org/.

**Europe (SHARE).** This is Version F in the harmonized files and incorporates the latest released version of SHARE data, release 8.0.0, which adds observations from Wave 8. It contains 139,620 observations or rows. It is a Respondent-level file so each row represents a unique Respondent. It also adds new variables and makes adjustments and corrections. However, we focus on data from SHARE Wave 6, with the release 8.0.0 as of February 2022. SHARE uses a multistage stratified sample. Its weighting variables make its data representative of the target populations in constituent countries. Wave 6 does not still have full coverage of European countries, with the following countries only added in Wave 7: Finland, Lithuania, Latvia, Slovakia, Romania, Bulgaria, Malta and Cyprus. Eligible cases leave us with a samples size of 66,708. SHARE provides person-level weights RwWTRESP, which are taken directly from SHARE’s respondent weight variables cciw_wW from Wave 1-6 and cciw_w7_REG for Wave 7, which are calibrated cross-sectional weights. Cross-sectional respondent weights are based on

the household design weight and the calibration variables of the respondent. Cross-sectional respondent weights are only generated for respondents age 50 or older. SHARE’s calibration is based on the size of each country’s populations of individuals born in 1964 or earlier across eight gender-age groups. Because we focus on individual level, we use the individual level respondent weights to account for non-response adjustment (variable “rwtresp”). We also use sampling weights to account for survey design. The sampling weights RwWTSAMP are taken directly from SHARE’s dw_wW variables, which are the sampling design weights at the individual and household levels. Because the inclusion probability of any eligible household member is the same as the inclusion probability of the whole household, these weights account for both the individual and the household. These weights are defined as the inverse of the probability of

being included in the sample at each wave, allowing users to compensate for unequal selection

probabilities of the sample units. See (<http://www.share-project.org>) for more details.

**China (CHARLS).** The China Health and Retirement Longitudinal Study (CHARLS) is a longitudinal study of individuals over age 45 in China. Version D incorporates the latest released version of CHARLS data, and adds variables for Wave 4. It contains 25,586 observations or rows. It is a Respondent-level file so each row represents a unique Respondent; The sample population was selected as part of a stratified, multistage probability design. We will use Wave 3. As we concentrate on ages 50 and above due to the other samples we do not include individuals younger than 50. The eligible number of interviews for Wave 3 amount to a sample size of 20,281. However, an important issue for doctor diagnosed diseases in Wave 3 of CHARLS is that respondents may be missing values due to an error in the Life History Survey code for "ever had" responses for doctor diagnosed health problems. Hence, after excluding these cases, we get a sample of 15,910 observations. The Harmonized CHARLS includes variables to allow users to produce weighted estimates with survey design adjusted standard errors where provided by CHARLS. Because we focus on individual level, we use the person level respondent weights to account for non-response adjustment (variable “rwtresp”). In addition to person-level weights, CHARLS uses a stratified (by per capita GDP of urban districts and rural counties) multistage (county/district-village/community-household) proportionate to population size (PPS) random sampling method to strictly control the quality of the samples. For more detail information about weight and survey design, please see http://charls.pku.edu.cn/en/page/documentation/2013_tracking-_survey at China Health and Retirement Longitudinal Study (CHARLS) website.

For more details on how the weights are constructed, refer to <https://hrsdata.isr.umich.edu/sites/default/files/documentation/data-descriptions/trk2018v2a.pdf>.

**KLOSA- Korea.** The Korean Longitudinal Study of Ageing (KLoSA) is a panel survey of people aged 45 and over and their partners, living in private households in Korea. The survey elicits information about demographics, income, assets, health, cognition, family structure and connections, health care use and costs, housing, job status and history, expectations, and insurance. KLoSA surveys respondents every two years. Funded by the Korean Ministry of Labor, the Korean Institute of Labor (KLI) collected the first two waves, and the Korea Employment Information Service (KEIS) collected the Waves 3, 4, 5 and 6 of KLoSA, with the first wave of the KLoSA survey being conducted in fall/winter of 2006. The sample population was selected as part of a stratified, multi-stage area probability design. The first component of this sampling framework is the probability proportional to size (PPS) systematic sampling of the 2005 (South Korean) Census enumeration districts after stratifying by the location (15 major metropolitan cities and provinces) and characteristic of the district (urban or rural, and apartment building or non-apartment dwelling). Households were selected within PSUs from a listing of households in the Census identified as age-eligible; that is, inhabited by at least one person 45 years of age and older. This initial sample included 10,254 respondents age 45 and over. The second wave was conducted in 2008 and had 8,688 respondents. The third wave was conducted in 2010 and had 7,920 respondents. The fourth wave was conducted in 2012 and had 7,486 respondents. There was no refresher sample in Waves two through four. In 2014, a refreshment sample of individuals born in 1962 or 1963 was drawn and it included 920 individuals, which were added to the 7,029 remaining core sample respondents for a total of 7,949 Wave 5 respondents. The sixth wave was conducted in 2016 and had 7,490 respondents. We will focus on Wave 5, hamornized Version C on ages >50, with a sample of 7,028. We use RwWTRESP, the person-level cross-sectional weight provided by KLoSA. The weight is provided to make the data a nationally representative sample. Starting in Wave 5, RwWTRESP takes the values of the cross-sectional weights constructed for the combined sample of the original cohort and the new Wave 5 cohort. In addition to weights, KLoSA also provides stratification and cluster variables to account for KLoSA’s complex survey design. The three region variables, 15 cities/provinces (region1), dong/eup/myeon (region2), and housing type (enu_type) were used for the stratification variable, and enumeration district (enu) was the cluster variable. Hence, we use RAECLUST as the clustering variable based off the primary sampling units in KLoSA (w01enu). These units correspond to the enumeration district included in the sample. An enumeration district is a unit defined by the National Statistics Office for the Korean Population and Housing Census. Of 261,237 enumeration districts in South Korea, 1,000 were selected for KLoSA sampling and are included in Wave 1. A new cohort of respondents was added in Wave 5. The clustering variable for those individuals is based on the Wave 5 sampling units (w05enu). Additionally, we use RAESTRAT, the stratification variable of the KLoSA data. It was created by concatenating variables w01region1, w01region2, and w01enu_type, which corresponded to a particular region or metropolitan city (Region 1), village or town status (Region 2), and whether or not the respondent lived in a house or apartment complex (W0wenu_type). The stratification variable for the new cohort of respondents added in Wave 5 is based on concatenating variables w05region1, w05region2, and w05enu_type.

For more detailed information about weight and survey design, please see the “KLoSA-2007_Wave1_userguide.pdf” at Korea Employment Information Service (KEIS) website (https://survey.keis.or.kr/eng/klosa/klosa01.jsp).

We focus on this specific set of countries as our aim is to have the most diverse group of nations while retaining the highest possible level of concordance across the harmonized health variables. Hence, we choose these countries and years due to the following specific reasons: 1. these are the available countries for which the highest possible concordance among surveys is available for health information; 2. these countries have unique epidemiological and mortality trajectories that include countries with fast-paced mortality transitions, such as Korea and slow pioneering countries like Sweden; 3. Different cultural backgrounds, gender norms, and health systems, which enable us to investigate whether specific gender patterns in inequality in health and mortality emerge in those settings. We focus on ages above 60 y to be coherent towards the definition of old age across countries. While most developed countries define old age as 65 y, for China and Mexico it is age 60 y. For more details on the data, refer to the Supplementary Information (SI) section on Materials and Table S3 for sample characteristics.

1. **Mortality data**

For mortality data, we use UN life tables from the 2022 Revision of World Population Prospects (United Nations 2022) for all countries with the exceptions of England, where the life tables are from the Office for National Statistics UK (ONS) estimates, as the ELSA study does not include Wales.


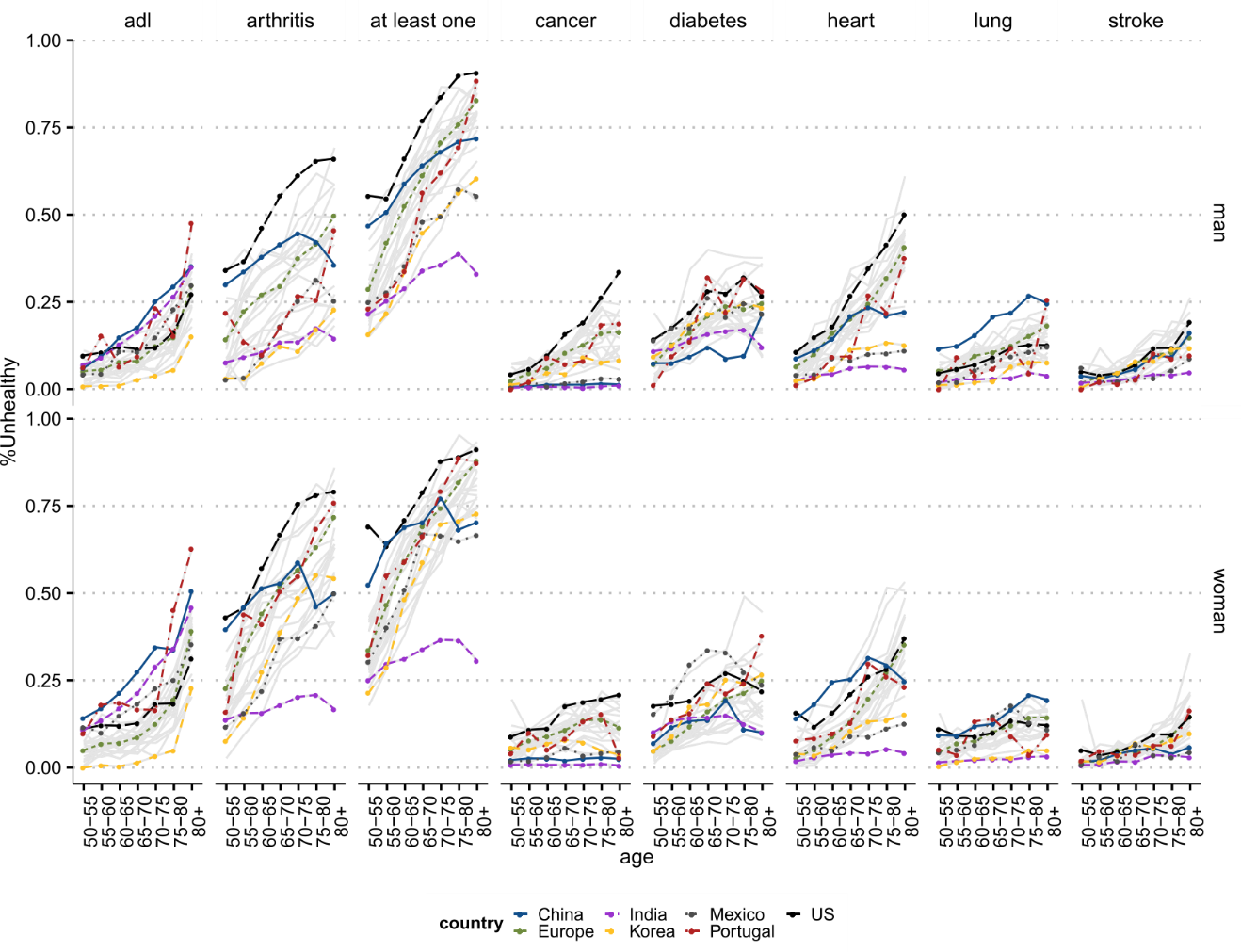


Fig. S1. Age-specific prevalence of health conditions for all countries (grey in the background) and selected countries for women and men. Notes. Panel “ADL” refers to the 5-item list of activities of daily living (ADLs), which include bathing, dressing, eating, getting in and out of bed, and using the toilet. Panel “At least one” refers to the constructed variable having at least one chronic doctor diagnosed diseases, which include diabetes, heart conditions, arthritis, cancer, stroke, and lung disease. Source: Gateway to Global Aging Data, Produced by the Program on Global Aging, Health & Policy, University of Southern California with funding from the National Institute on Aging (R01 AG030153).


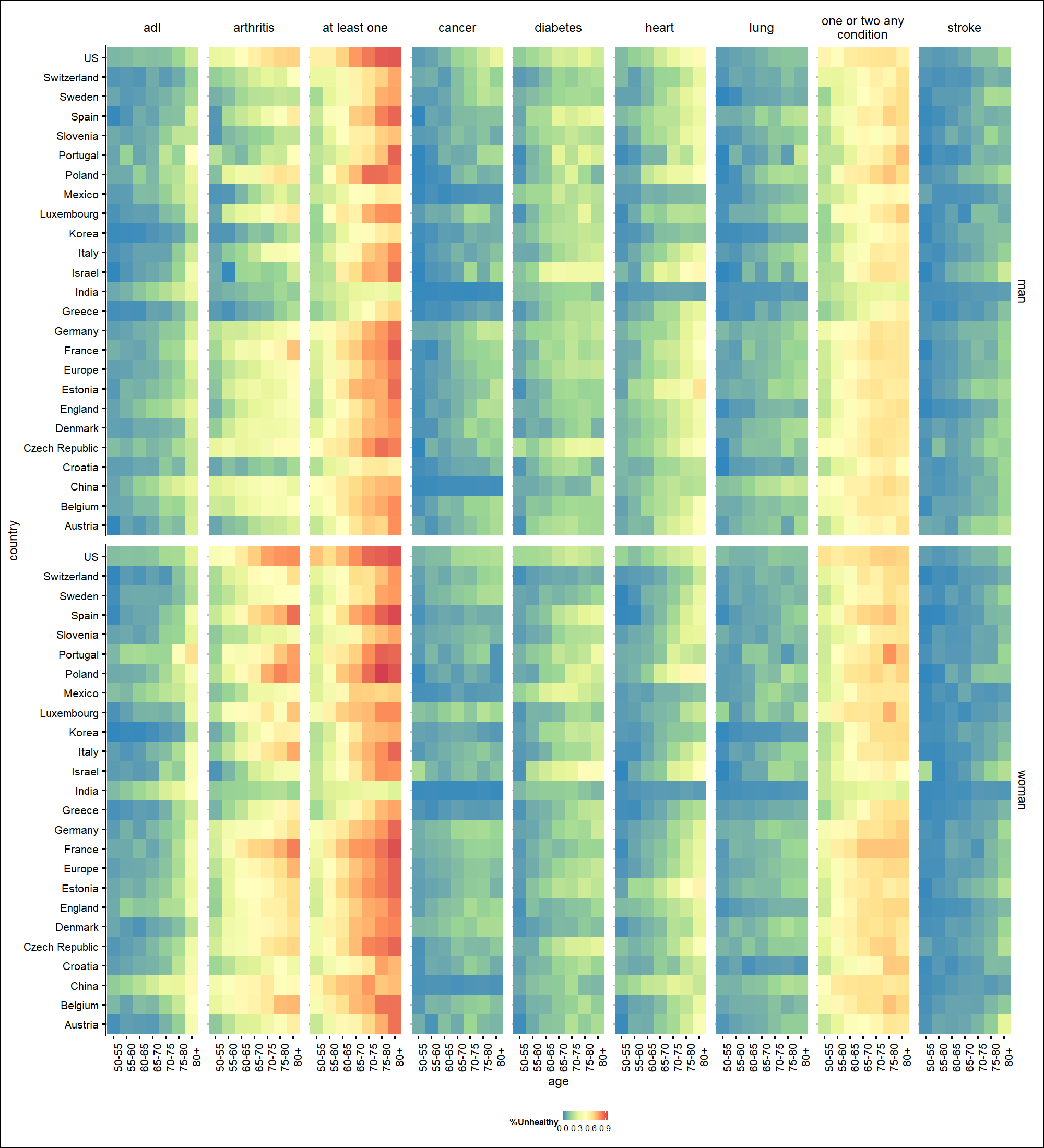


Fig. S2. Age-specific prevalence heatmap of health conditions for all countries for women and men. Notes. Panel “ADL” refers to the 5-item list of activities of daily living (ADLs), which include bathing, dressing, eating, getting in and out of bed, and using the toilet. Panel “At least one” refers to the constructed variable having at least one chronic doctor diagnosed diseases, which include diabetes, heart conditions, arthritis, cancer, stroke, and lung disease. Panel “One or two any condition” is similar, but aims at describing co-morbidities. Source: Gateway to Global Aging Data, Produced by the Program on Global Aging, Health & Policy, University of Southern California with funding from the National Institute on Aging (R01 AG030153).


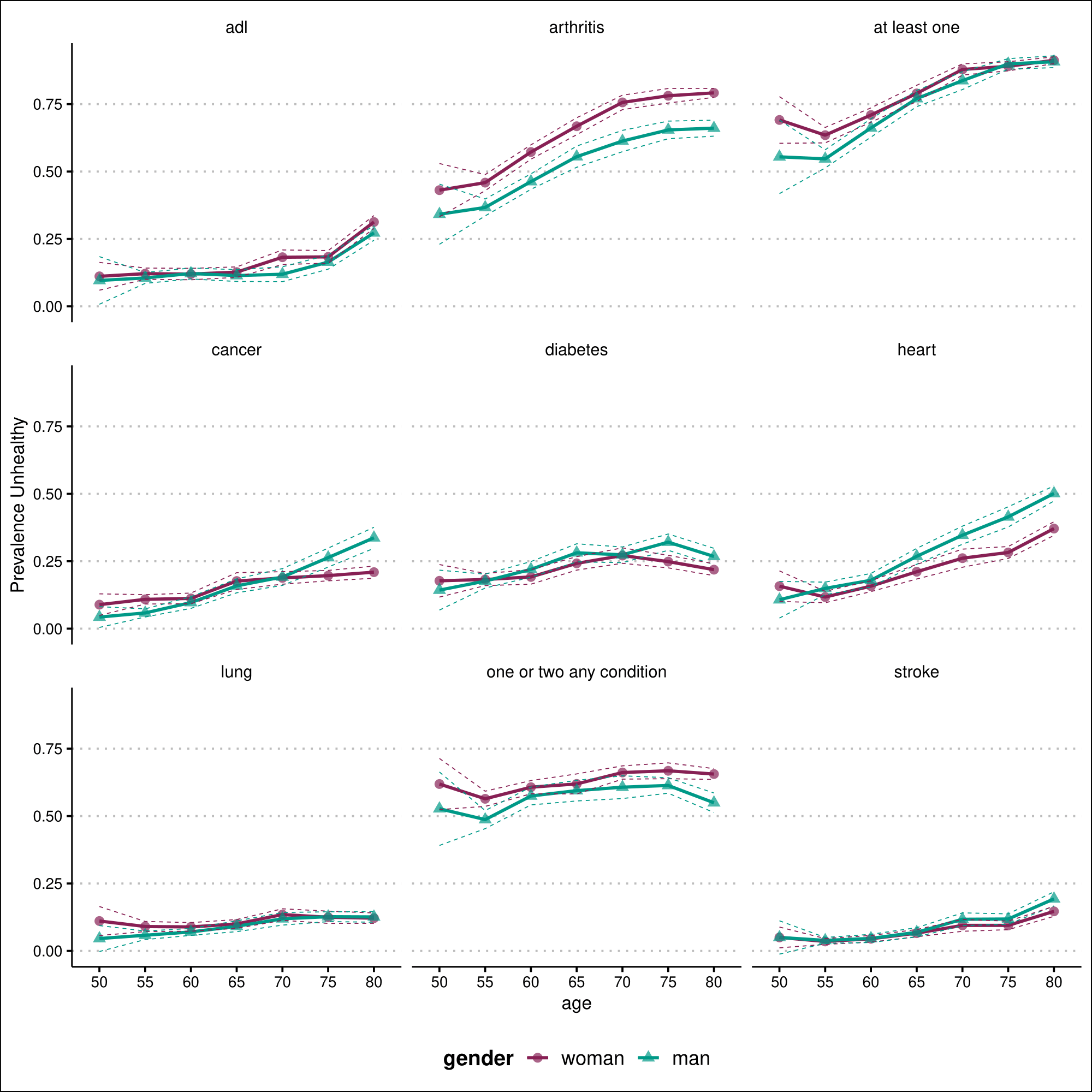


Fig. S3. Age-specific prevalence of health conditions for US (HRS), for women and men. Notes. Panel “ADL” refers to the 5-item list of activities of daily living (ADLs), which include bathing, dressing, eating, getting in and out of bed, and using the toilet. Panel “At least one” refers to the constructed variable having at least one chronic doctor diagnosed diseases, which include diabetes, heart conditions, arthritis, cancer, stroke, and lung disease. Panel “One or two any condition” is similar, but aims at describing co-morbidities. Source: Gateway to Global Aging Data, Produced by the Program on Global Aging, Health & Policy, University of Southern California with funding from the National Institute on Aging (R01 AG030153).


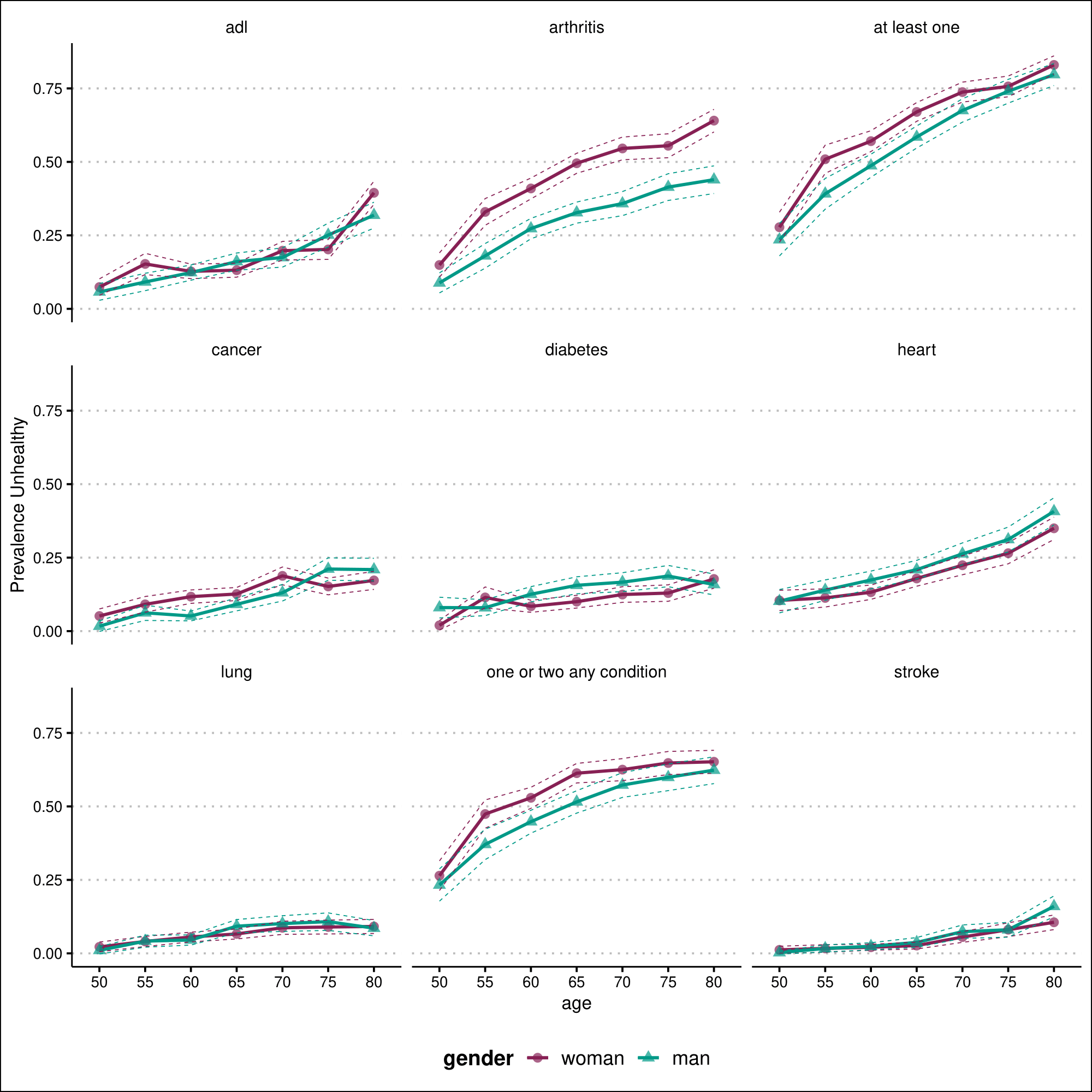


Fig. S4. Age-specific prevalence of health conditions for England (ELSA), for women and men. Notes. Panel “ADL” refers to the 5-item list of activities of daily living (ADLs), which include bathing, dressing, eating, getting in and out of bed, and using the toilet. Panel “At least one” refers to the constructed variable having at least one chronic doctor diagnosed diseases, which include diabetes, heart conditions, arthritis, cancer, stroke, and lung disease. Panel “One or two any condition” is similar, but aims at describing co-morbidities. Source: Gateway to Global Aging Data, Produced by the Program on Global Aging, Health & Policy, University of Southern California with funding from the National Institute on Aging (R01 AG030153).


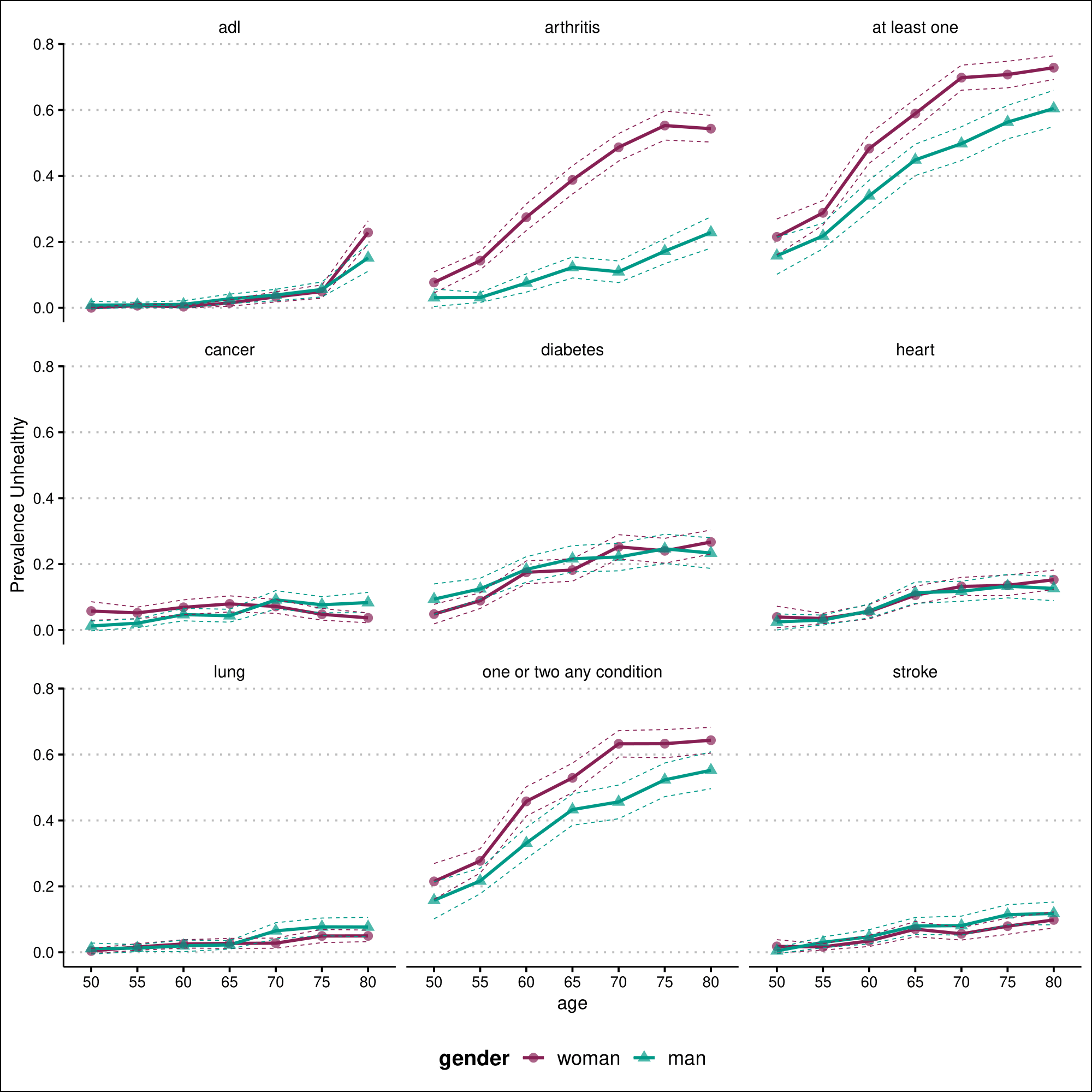


Fig. S5. Age-specific prevalence of health conditions for Korea (KLOSA), for women and men. Notes. Panel “ADL” refers to the 5-item list of activities of daily living (ADLs), which include bathing, dressing, eating, getting in and out of bed, and using the toilet. Panel “At least one” refers to the constructed variable having at least one chronic doctor diagnosed diseases, which include diabetes, heart conditions, arthritis, cancer, stroke, and lung disease. Panel “One or two any condition” is similar, but aims at describing co-morbidities. Source: Gateway to Global Aging Data, Produced by the Program on Global Aging, Health & Policy, University of Southern California with funding from the National Institute on Aging (R01 AG030153).


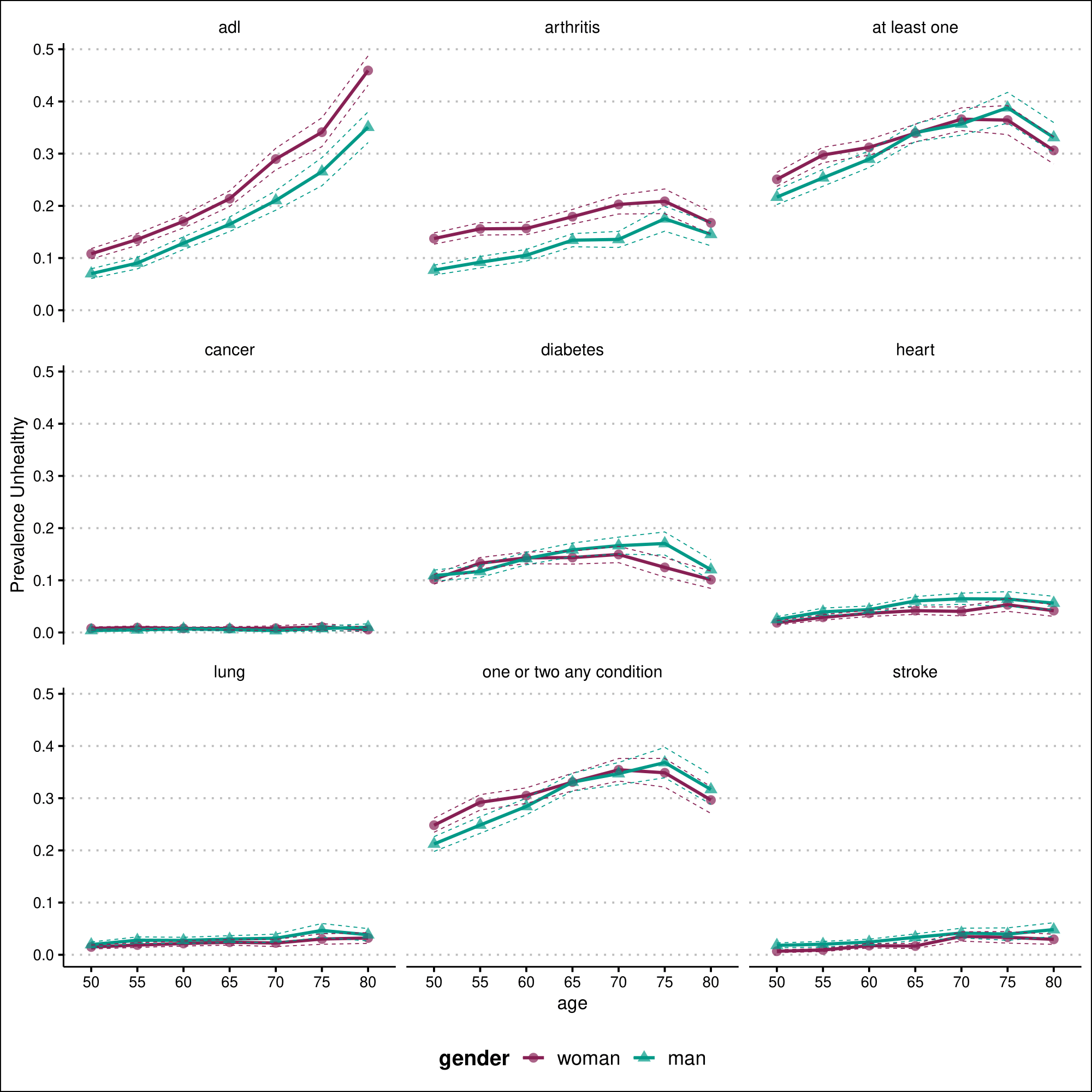


Fig. S6. Age-specific prevalence of health conditions for India (LASI), for women and men. Notes. Panel “ADL” refers to the 5-item list of activities of daily living (ADLs), which include bathing, dressing, eating, getting in and out of bed, and using the toilet. Panel “At least one” refers to the constructed variable having at least one chronic doctor diagnosed diseases, which include diabetes, heart conditions, arthritis, cancer, stroke, and lung disease. Panel “One or two any condition” is similar, but aims at describing co-morbidities. Source: Gateway to Global Aging Data, Produced by the Program on Global Aging, Health & Policy, University of Southern California with funding from the National Institute on Aging (R01 AG030153).


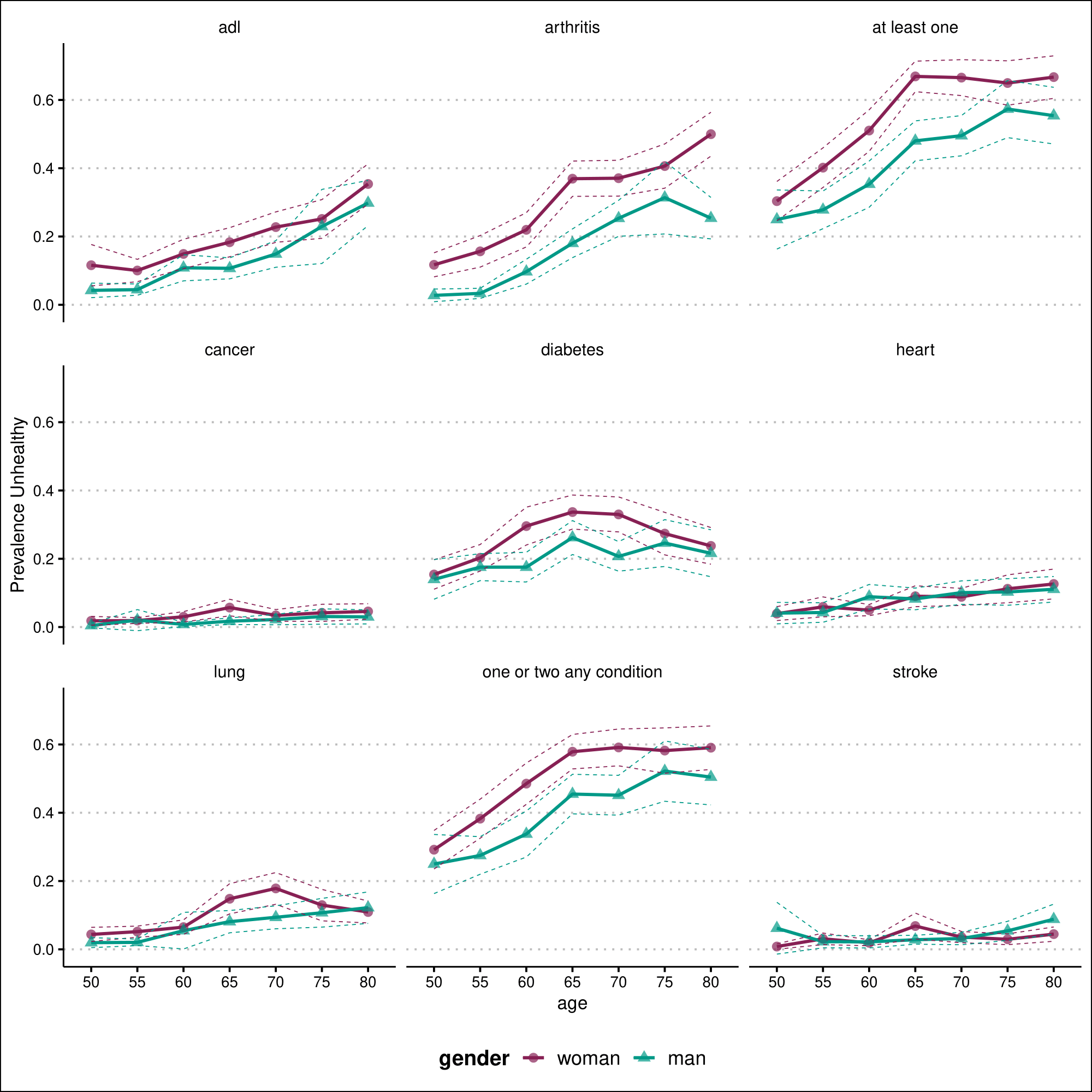


Fig. S7. Age-specific prevalence of health conditions for Mexico (MHAS), for women and men. Notes. Panel “ADL” refers to the 5-item list of activities of daily living (ADLs), which include bathing, dressing, eating, getting in and out of bed, and using the toilet. Panel “At least one” refers to the constructed variable having at least one chronic doctor diagnosed diseases, which include diabetes, heart conditions, arthritis, cancer, stroke, and lung disease. Panel “One or two any condition” is similar, but aims at describing co-morbidities. Source: Gateway to Global Aging Data, Produced by the Program on Global Aging, Health & Policy, University of Southern California with funding from the National Institute on Aging (R01 AG030153).


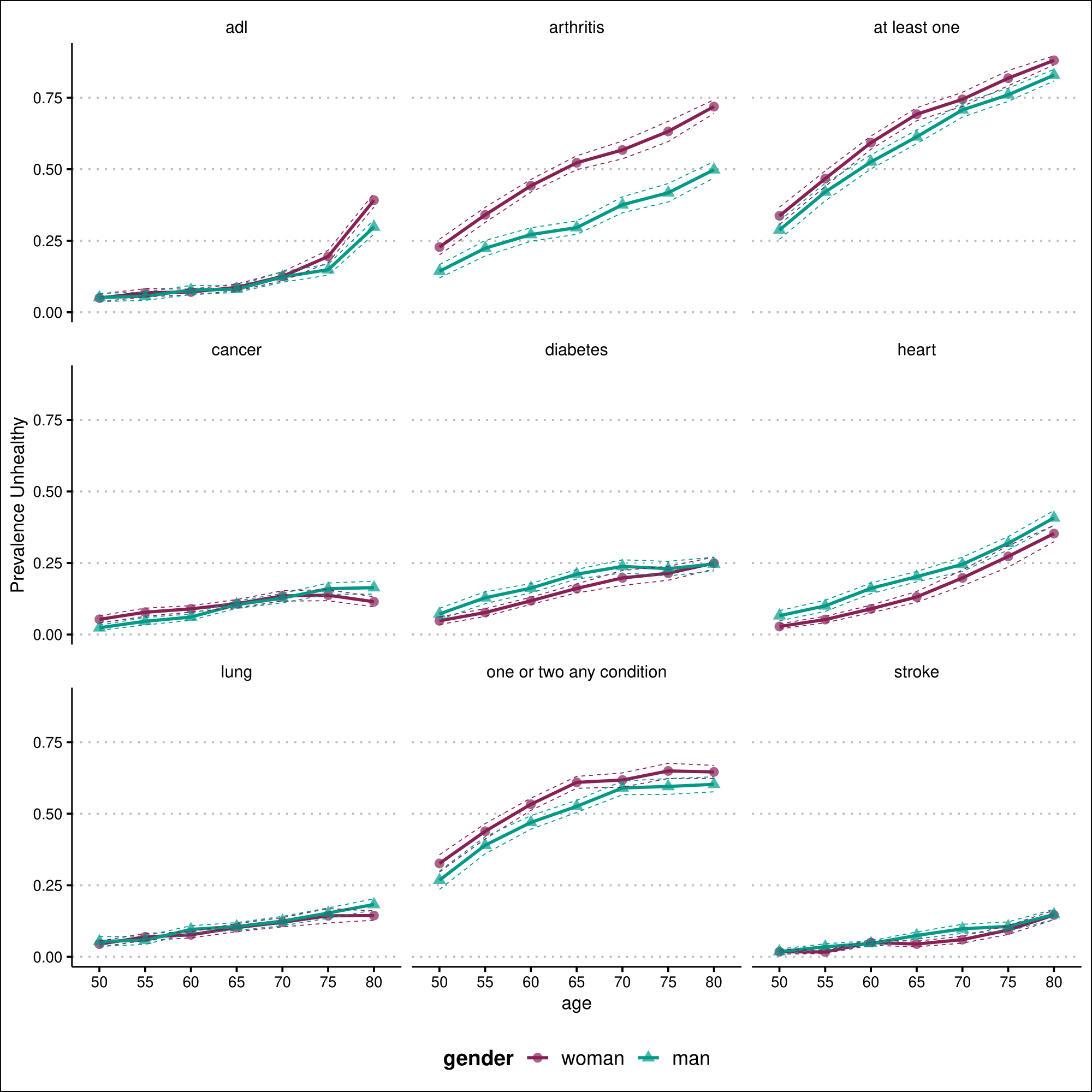


Fig. S8. Age-specific prevalence of health conditions for Europe (SHARE), for women and men. Notes. Panel “ADL” refers to the 5-item list of activities of daily living (ADLs), which include bathing, dressing, eating, getting in and out of bed, and using the toilet. Panel “At least one” refers to the constructed variable having at least one chronic doctor diagnosed diseases, which include diabetes, heart conditions, arthritis, cancer, stroke, and lung disease. Panel “One or two any condition” is similar, but aims at describing co-morbidities. Source: Gateway to Global Aging Data, Produced by the Program on Global Aging, Health & Policy, University of Southern California with funding from the National Institute on Aging (R01 AG030153).


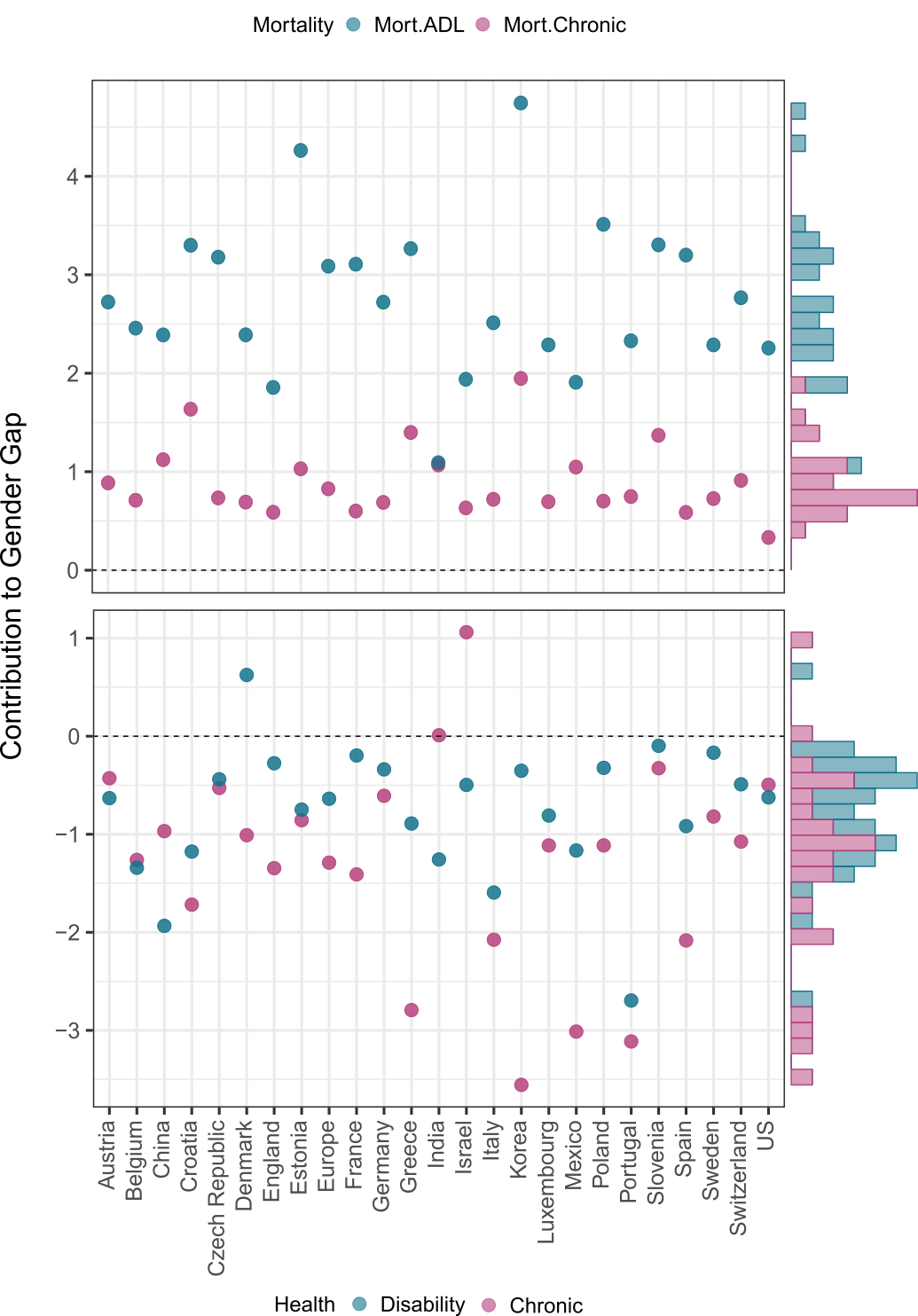


Fig. S9. Mortality and health components and their contribution to explaining the total gender gap. Source: Gateway to Global Aging Data, Produced by the Program on Global Aging, Health & Policy, University of Southern California with funding from the National Institute on Aging (R01 AG030153

**Table S2.** Absolute total life expectancy and disability-free life expectancy (DFLE), by gender

| Country | Life expectancy | | | Disability-Free Life Expectancy (DFLE) | | | | | | |
| --- | --- | --- | --- | --- | --- | --- | --- | --- | --- | --- |
|  | women | men | GAP (w-m) | women | 95% CI | | men | 95% CI | | GAP (w-m) |
|  |  |  |  |  | lower | upper |  | lower | upper |  |
| US | 24.84 | 21.85 | 2.99 | 19.92 | 20.49 | 19.35 | 18.29 | 18.83 | 17.75 | 1.63 |
| China | 23.06 | 19.25 | 3.81 | 15.18 | 16.17 | 14.20 | 14.73 | 15.63 | 13.83 | 0.45 |
| Mexico | 22.30 | 19.66 | 2.64 | 17.02 | 18.11 | 15.92 | 16.27 | 17.31 | 15.24 | 0.74 |
| India | 19.48 | 17.86 | 1.63 | 13.94 | 14.33 | 13.55 | 14.11 | 14.44 | 13.77 | -0.17 |
| Korea | 27.13 | 21.57 | 5.56 | 24.70 | 25.22 | 24.17 | 20.30 | 20.76 | 19.85 | 4.39 |
| England | 25.40 | 22.73 | 2.68 | 19.57 | 20.36 | 18.77 | 17.99 | 18.77 | 17.20 | 1.58 |
| Europe |  |  |  |  |  |  |  |  |  |  |
| *(Pooled)* | 24.07 | 19.92 | 4.15 | 19.51 | 19.92 | 19.10 | 17.06 | 17.41 | 16.70 | 2.45 |
| Austria | 25.52 | 21.82 | 3.70 | 21.07 | 22.06 | 20.07 | 18.98 | 19.92 | 18.03 | 2.09 |
| Belgium | 25.44 | 21.91 | 3.53 | 19.31 | 20.26 | 18.37 | 18.20 | 19.09 | 17.31 | 1.12 |
| Croatia | 22.59 | 18.32 | 4.28 | 18.36 | 19.78 | 16.93 | 16.23 | 17.15 | 15.31 | 2.12 |
| Czech Republic | 23.53 | 19.37 | 4.17 | 19.20 | 20.22 | 18.18 | 16.46 | 17.37 | 15.55 | 2.74 |
| Denmark | 24.80 | 21.81 | 2.99 | 21.96 | 22.71 | 21.20 | 18.94 | 20.52 | 17.36 | 3.01 |
| Estonia | 24.19 | 18.54 | 5.65 | 19.34 | 20.07 | 18.61 | 15.82 | 16.42 | 15.23 | 3.51 |
| France | 27.47 | 22.94 | 4.53 | 21.63 | 22.80 | 20.46 | 18.72 | 19.78 | 17.66 | 2.91 |
| Germany | 25.10 | 21.46 | 3.64 | 20.63 | 21.77 | 19.48 | 18.24 | 19.16 | 17.33 | 2.38 |
| Greece | 25.34 | 21.33 | 4.01 | 21.82 | 22.68 | 20.97 | 19.45 | 20.15 | 18.75 | 2.37 |
| Israel | 25.75 | 22.95 | 2.80 | 20.60 | 22.12 | 19.07 | 19.15 | 20.50 | 17.80 | 1.44 |
| Italy | 26.37 | 22.87 | 3.51 | 20.95 | 22.00 | 19.90 | 20.03 | 20.84 | 19.22 | 0.92 |
| Luxembourg | 25.65 | 22.59 | 3.07 | 21.10 | 22.72 | 19.48 | 19.62 | 20.72 | 18.52 | 1.48 |
| Poland | 23.86 | 18.84 | 5.01 | 18.40 | 19.62 | 17.18 | 15.21 | 16.16 | 14.27 | 3.19 |
| Portugal | 26.02 | 21.87 | 4.15 | 16.92 | 20.41 | 13.43 | 17.28 | 19.82 | 14.75 | -0.37 |
| Slovenia | 25.13 | 20.82 | 4.31 | 20.89 | 21.89 | 19.89 | 17.68 | 18.63 | 16.74 | 3.21 |
| Spain | 27.08 | 22.72 | 4.37 | 21.85 | 23.02 | 20.67 | 19.56 | 20.63 | 18.49 | 2.28 |
| Sweden | 25.77 | 23.03 | 2.73 | 22.74 | 23.68 | 21.80 | 20.62 | 21.43 | 19.80 | 2.12 |
| Switzerland | 26.62 | 23.36 | 3.26 | 23.74 | 24.86 | 22.63 | 21.47 | 22.34 | 20.59 | 2.28 |

Source: Gateway to Global Aging Data, Produced by the Program on Global Aging, Health & Policy, University of Southern California with funding from the National Institute on Aging (R01 AG03015)

Table S3. Absolute total life expectancy and chronic-free life expectancy (CFLE), by gender

| Country | Life expectancy | | | Chronic-Free life expectancy (CFLE) | | | | | | |
| --- | --- | --- | --- | --- | --- | --- | --- | --- | --- | --- |
|  | women | men | GAP (w-m) | women | 95% CI | | men | 95% CI | | GAP (w-m) |
|  |  |  |  |  | lower | upper |  | lower | upper |  |
| US | 24.84 | 21.85 | 2.99 | 3.97 | 4.48 | 3.45 | 4.13 | 4.74 | 3.52 | -0.16 |
| China | 23.06 | 19.25 | 3.81 | 6.68 | 7.62 | 5.74 | 6.53 | 7.40 | 5.66 | 0.15 |
| Mexico | 22.30 | 19.66 | 2.64 | 8.23 | 9.50 | 6.96 | 10.20 | 11.56 | 8.84 | -1.97 |
| India | 19.48 | 17.86 | 1.63 | 12.94 | 13.34 | 12.53 | 11.86 | 12.24 | 11.48 | 1.08 |
| Korea | 27.13 | 21.57 | 5.56 | 9.48 | 10.56 | 8.39 | 11.08 | 12.17 | 10.00 | -1.61 |
| England | 25.40 | 22.73 | 2.68 | 7.05 | 7.90 | 6.21 | 7.81 | 8.69 | 6.93 | -0.76 |
| Europe |  |  |  |  |  |  |  |  |  |  |
| *(Pooled)* | 24.07 | 19.92 | 4.15 | 5.98 | 6.51 | 5.46 | 6.45 | 6.91 | 5.98 | -0.46 |
| Austria | 25.52 | 21.82 | 3.70 | 8.39 | 9.80 | 6.97 | 7.93 | 9.42 | 6.44 | 0.46 |
| Belgium | 25.44 | 21.91 | 3.53 | 6.09 | 7.16 | 5.01 | 6.64 | 7.71 | 5.57 | -0.55 |
| Croatia | 22.59 | 18.32 | 4.28 | 9.02 | 10.71 | 7.34 | 9.10 | 10.68 | 7.53 | -0.08 |
| Czech Republic | 23.53 | 19.37 | 4.17 | 5.42 | 6.42 | 4.41 | 5.21 | 6.35 | 4.07 | 0.21 |
| Denmark | 24.80 | 21.81 | 2.99 | 6.77 | 7.60 | 5.93 | 7.09 | 8.08 | 6.09 | -0.32 |
| Estonia | 24.19 | 18.54 | 5.65 | 5.40 | 6.07 | 4.74 | 5.23 | 5.77 | 4.69 | 0.17 |
| France | 27.47 | 22.94 | 4.53 | 4.91 | 6.13 | 3.68 | 5.71 | 6.97 | 4.46 | -0.81 |
| Germany | 25.10 | 21.46 | 3.64 | 6.55 | 7.85 | 5.25 | 6.47 | 7.63 | 5.30 | 0.08 |
| Greece | 25.34 | 21.33 | 4.01 | 9.98 | 11.23 | 8.72 | 11.37 | 12.49 | 10.25 | -1.39 |
| Israel | 25.75 | 22.95 | 2.80 | 8.46 | 10.59 | 6.32 | 6.77 | 8.79 | 4.74 | 1.69 |
| Italy | 26.37 | 22.87 | 3.51 | 7.19 | 8.43 | 5.94 | 8.54 | 9.75 | 7.33 | -1.36 |
| Luxembourg | 25.65 | 22.59 | 3.07 | 6.72 | 8.36 | 5.07 | 7.13 | 8.97 | 5.30 | -0.42 |
| Poland | 23.86 | 18.84 | 5.01 | 4.11 | 5.35 | 2.87 | 4.52 | 5.78 | 3.26 | -0.41 |
| Portugal | 26.02 | 21.87 | 4.15 | 6.00 | 8.89 | 3.12 | 8.37 | 11.28 | 5.46 | -2.37 |
| Slovenia | 25.13 | 20.82 | 4.31 | 9.68 | 11.00 | 8.36 | 8.64 | 9.89 | 7.39 | 1.04 |
| Spain | 27.08 | 22.72 | 4.37 | 5.08 | 6.35 | 3.81 | 6.57 | 7.93 | 5.21 | -1.50 |
| Sweden | 25.77 | 23.03 | 2.73 | 9.00 | 10.26 | 7.74 | 9.09 | 10.35 | 7.82 | -0.09 |
| Switzerland | 26.62 | 23.36 | 3.26 | 9.23 | 10.95 | 7.50 | 9.39 | 10.85 | 7.92 | -0.16 |

Source: Gateway to Global Aging Data, Produced by the Program on Global Aging, Health & Policy, University of Southern California with funding from the National Institute on Aging (R01 AG03015)

Table S4. Decomposition of the gender gap (women-men) in disability-free life expectancy (DFLE) ages 60 y and over into mortality and disability effects by country, with 95% confidence intervals.

| Country | LE | DFLE | 95%CI | Components | | | |
| --- | --- | --- | --- | --- | --- | --- | --- |
|  |  |  |  | Mortality | 95%CI | Disability | 95%CI |
|  |  |  |  |  |  |  |  |
| US | 2.99 | 1.63 | [1.60, 1.67] | 2.26 | [2.18, 2.33] | -0.62 | [-0.58, -0.66] |
| China | 3.81 | 0.45 | [0.37, 0.54] | 2.39 | [2.14, 2.64] | -1.93 | [-1.77, -2.10] |
| Mexico | 2.64 | 0.74 | [0.68, 0.80] | 1.91 | [1.75, 2.07] | -1.17 | [-1.07, -1.26] |
| India | 1.63 | -0.17 | [-0.22, 0.1] | 1.09 | [1.05, 1.13] | -1.26 | [-1.27, -1.25] |
| Korea | 5.56 | 4.39 | [4.33, 4.46] | 4.74 | [4.57, 4.93] | -0.35 | [-0.24, -0.46] |
| England | 2.68 | 1.58 | [1.57, 1.60] | 1.86 | [1.75, 1.96] | -0.28 | [-0.19, -0.37] |
| Europe |  |  |  |  |  |  |  |
| (*Pooled*) | 4.15 | 2.45 | [2.39, 2.50] | 3.09 | [3.00, 3.17] | -0.64 | [-0.60, -0.67] |
| Austria | 3.7 | 2.09 | [2.04, 2.14] | 2.72 | [2.52, 2.93] | -0.63 | [-0.48, -0.79] |
| Belgium | 3.53 | 1.12 | [1.06, 1.17] | 2.46 | [2.30, 2.62] | -1.34 | [-1.24, -1.45] |
| Croatia | 4.28 | 2.12 | [1.62, 2.63] | 3.3 | [2.98, 3.62] | -1.18 | [-1.36, -0.99] |
| Czechia | 4.17 | 2.74 | [2.63, 2.85] | 3.18 | [2.94, 3.41] | -0.44 | [-0.31, -0.56] |
| Denmark | 2.99 | 3.01 | [2.19, 3.84] | 2.39 | [2.13, 2.65] | 0.62 | [1.71, -0.46] |
| Estonia | 5.65 | 3.51 | [3.38, 3.65] | 4.26 | [4.03, 4.49] | -0.75 | [-0.65, -0.85] |
| France | 4.53 | 2.91 | [2.80, 3.02] | 3.11 | [2.87, 3.35] | -0.2 | [-0.06, -0.33] |
| Germany | 3.64 | 2.38 | [2.16, 2.61] | 2.72 | [2.51, 2.93] | -0.34 | [-0.35, -0.32] |
| Greece | 4.01 | 2.37 | [2.22, 2.53] | 3.27 | [3.10, 3.44] | -0.89 | [-0.88, -0.91] |
| Israel | 2.8 | 1.44 | [1.27, 1.61] | 1.94 | [1.72, 2.16] | -0.50 | [-0.45, -0.55] |
| Italy | 3.51 | 0.92 | [0.68, 1.16] | 2.51 | [2.33, 2.69] | -1.59 | [-1.66, -1.53] |
| Luxembourg | 3.07 | 1.48 | [0.96, 2.00] | 2.29 | [2.08, 2.50] | -0.81 | [-1.12, -0.49] |
| Poland | 5.01 | 3.19 | [2.91, 3.47] | 3.51 | [3.17, 3.86] | -0.32 | [-0.25, -0.39] |
| Portugal | 4.15 | -0.37 | [-1.32,0.59] | 2.33 | [1.64, 3.02] | -2.70 | [-2.97, -2.42] |
| Slovenia | 4.31 | 3.21 | [3.15, 3.26] | 3.3 | [3.08, 3.53] | -0.10 | [0.07, -0.27] |
| Spain | 4.37 | 2.28 | [2.18, 2.39] | 3.2 | [2.97, 3.43] | -0.92 | [-0.80, -1.04] |
| Sweden | 2.73 | 2.12 | [2.00, 2.24] | 2.29 | [2.17, 2.41] | -0.17 | [-0.17, -0.16] |
| Switzerland | 3.26 | 2.28 | [2.03, 2.52] | 2.77 | [2.59, 2.95] | -0.49 | [-0.55, -0.43] |
|  | | | | | | | |

Source: Gateway to Global Aging Data, Produced by the Program on Global Aging, Health & Policy, University of Southern California with funding from the National Institute on Aging (R01 AG03015)

Table S5. Decomposition of the gender gap (women-men) in chronic disease-free life expectancy (CFLE) at ages 60 y and over into mortality and chronic effects by country, with 95% confidence intervals.

| Country | LE | CFLE | 95%CI | Components | | | |
| --- | --- | --- | --- | --- | --- | --- | --- |
|  |  |  |  | Mortality | 95%CI | Chronic | 95%CI |
|  |  |  |  |  |  |  |  |
| US | 2.99 | -0.16 | [-0.07, -0.26] | 0.33 | [0.27, 0.39] | -0.49 | [-0.34, -0.65] |
| China | 3.81 | 0.15 | [0.08, 0.23] | 1.12 | [0.91, 1.34] | -0.97 | [-0.82, -1.11] |
| Mexico | 2.64 | -1.97 | [-1.87, -2.06] | 1.05 | [0.87, 1.23] | -3.01 | [-2.74, -3.29] |
| India | 1.63 | 1.08 | [1.06, 1.10] | 1.07 | [1.03, 1.11] | 0.01 | [0.03, -0.01] |
| Korea | 5.56 | -1.61 | [-1.61, -1.61] | 1.95 | [1.70, 2.20] | -3.56 | [-3.31, -3.80] |
| England | 2.68 | -0.76 | [-0.72, -0.79] | 0.59 | [0.49, 0.68] | -1.35 | [-1.22, -1.47] |
| Europe |  |  |  |  |  |  |  |
| (*Pooled*) | 4.15 | -0.46 | [-0.52, -0.40] | 0.83 | [0.74, 0.91] | -1.29 | [-1.27, -1.31] |
| Austria | 3.7 | 0.46 | [0.53, 0.39] | 0.89 | [0.66, 1.11] | -0.43 | [-0.13, -0.73] |
| Belgium | 3.53 | -0.55 | [-0.56, -0.55] | 0.71 | [0.56, 0.86] | -1.26 | [-1.12, -1.41] |
| Croatia | 4.28 | -0.08 | [-0.19, 0.03] | 1.64 | [1.25, 2.02] | -1.72 | [-1.44, -1.99] |
| Czechia | 4.17 | 0.21 | [0.34, 0.07] | 0.73 | [0.51, 0.96] | -0.53 | [-0.16, -0.89] |
| Denmark | 2.99 | -0.32 | [-0.16, -0.48] | 0.69 | [0.62, 0.77] | -1.01 | [-0.77, -1.25] |
| Estonia | 5.65 | 0.17 | [0.05, 0.30] | 1.03 | [0.90, 1.16] | -0.86 | [-0.85, -0.86] |
| France | 4.53 | -0.81 | [-0.78, -0.84] | 0.6 | [0.42, 0.78] | -1.41 | [-1.20, -1.62] |
| Germany | 3.64 | 0.08 | [-0.05, 0.22] | 0.69 | [0.50, 0.88] | -0.61 | [-0.55, -0.66] |
| Greece | 4.01 | -1.39 | [-1.53, -1.26] | 1.4 | [1.19, 1.61] | -2.79 | [-2.71, -2.87] |
| Israel | 2.8 | 1.69 | [1.58, 1.81] | 0.63 | [0.42, 0.84] | 1.06 | [1.16, 0.96] |
| Italy | 3.51 | -1.36 | [-1.39, -1.32] | 0.72 | [0.55, 0.89] | -2.08 | [-1.94, -2.21] |
| Luxembourg | 3.07 | -0.42 | [-0.23, -0.61] | 0.7 | [0.52, 0.87] | -1.11 | [-0.75, -1.48] |
| Poland | 5.01 | -0.41 | [-0.39, -0.43] | 0.7 | [0.43, 0.97] | -1.11 | [-0.82, -1.40] |
| Portugal | 4.15 | -2.37 | [-2.34, -2.39] | 0.75 | [0.31, 1.19] | -3.11 | [-2.65, -3.58] |
| Slovenia | 4.31 | 1.04 | [0.98, 1.11] | 1.37 | [1.12, 1.62] | -0.33 | [-0.14, -0.51] |
| Spain | 4.37 | -1.5 | [-1.41, -1.58] | 0.59 | [0.41, 0.77] | -2.08 | [-1.82, -2.35] |
| Sweden | 2.73 | -0.09 | [-0.09, -0.10] | 0.73 | [0.59, 0.87] | -0.82 | [-0.67, -0.96] |
| Switzerland | 3.26 | -0.16 | [-0.43, 0.10] | 0.91 | [0.71, 1.11] | -1.07 | [-1.14, -1.01] |

Source: Gateway to Global Aging Data, Produced by the Program on Global Aging, Health & Policy, University of Southern California with funding from the National Institute on Aging (R01 AG03015)

Table S3. Survey Characteristics

| **Variable** | **China (CHARLS) WAVE 3** | | | **USA (HRS) WAVE 12** | | | **Mexico (MHAS) WAVE 4** | | | | | **India (LASI) WAVE 1*** | | | | | | **Korea (KLoSA) WAVE 5** | | | | | | **England (ELSA) WAVE 7** | | | | | | | **Europe (SHARE) WAVE 6 **** | | | |
| --- | --- | --- | --- | --- | --- | --- | --- | --- | --- | --- | --- | --- | --- | --- | --- | --- | --- | --- | --- | --- | --- | --- | --- | --- | --- | --- | --- | --- | --- | --- | --- | --- | --- | --- |
|  | **woman**, N = 199,073,750^1^ | **man**, N = 192,363,273^1^ | p-value^2^ | **woman**, N = 46,430,206^1^ | **man**, N = 39,363,205^1^ | p-value^2^ | **woman**, N = 10,754,392^1^ | **man**, N = 8,591,521^1^ | p-value^2^ | | **woman**, N = 27,072^1^ | | **man**, N = 27,211^1^ | | p-value^2^ | | **woman**, N = 7,653,018^1^ | | **man**, N = 6,512,376^1^ | | p-value^2^ | | **woman**, N = 4,273^1^ | | **man**, N = 3,878^1^ | | p-value^2^ | | **woman**, N = 80,385,989^1^ | | | **man**, N = 67,940,337^1^ | p-value^2^ |  |
| **ADLs** |  |  | **<0.001** |  |  | **<0.001** |  |  | **<0.001** | |  | |  | | **<0.001** | |  | |  | | **0.003** | |  | |  | | **0.057** | |  | | |  | **<0.001** |  |
| no disability | 150,893,743 (75.8%) | 161,391,539 (83.9%) |  | 38,871,400 (83.7%) | 34,046,381 (86.5%) |  | 8,944,390 (83.2%) | 7,601,903 (88.5%) |  | | 21,828 (80.6%) | | 23,454 (86.2%) | |  | | 7,344,536 (96.0%) | | 6,332,573 (97.2%) | |  | | 3,526 (82.5%) | | 3,304 (85.2%) | |  | | 68,775,998 (85.6%) | | | 60,737,519 (89.4%) |  |  |
| yes | 48,180,008 (24.2%) | 30,971,734 (16.1%) |  | 7,558,806 (16.3%) | 5,316,824 (13.5%) |  | 1,810,002 (16.8%) | 989,618 (11.5%) |  | | 5,245 (19.4%) | | 3,757 (13.8%) | |  | | 308,482 (4.0%) | | 179,803 (2.8%) | |  | | 747 (17.5%) | | 574 (14.8%) | |  | | 11,609,992 (14.4%) | | | 7,202,818 (10.6%) |  |  |
| **Age** | 63 (10) | 63 (9) | **0.200** | 67 (10) | 66 (9) | **<0.001** | 64 (9) | 65 (9) | **0.007** | | 62 (9) | | 62 (9) | | **0.005** | | 66 (10) | | 64 (9) | | **<0.001** | |  | |  | |  | | 67 (11) | | | 65 (10) | **<0.001** |  |
| **Chronic diseases** |  |  | **<0.001** |  |  | **<0.001** |  |  | **<0.001** | |  | |  | | **<0.001** | |  | |  | | **<0.001** | |  | |  | | **0.009** | |  | | |  | **<0.001** |  |
| no chronic | 68,432,541 (34.4%) | 79,570,990 (41.4%) |  | 10,537,872 (22.7%) | 11,075,867 (28.1%) |  | 5,226,551 (48.6%) | 5,186,315 (60.4%) |  | | 18,768 (69.3%) | | 19,441 (71.4%) | |  | | 3,794,099 (49.6%) | | 4,202,676 (64.5%) | |  | | 1,735 (40.6%) | | 1,896 (48.9%) | |  | | 29,197,080 (36.3%) | | | 30,180,372 (44.4%) |  |  |
| yes | 130,641,209 (65.6%) | 112,792,283 (58.6%) |  | 35,892,334 (77.3%) | 28,287,338 (71.9%) |  | 5,527,841 (51.4%) | 3,405,206 (39.6%) |  | | 8,305 (30.7%) | | 7,770 (28.6%) | |  | | 3,858,919 (50.4%) | | 2,309,700 (35.5%) | |  | | 2,538 (59.4%) | | 1,983 (51.1%) | |  | | 51,188,910 (63.7%) | | | 37,759,965 (55.6%) |  |  |
| **Heart** |  |  | **<0.001** |  |  | **<0.001** |  |  | **0.600** | |  | |  | | **<0.001** | |  | |  | | **0.062** | |  | |  | | **0.120** | |  | | |  | **<0.001** |  |
| no chronic | 154,660,188 (77.7%) | 162,066,797 (84.3%) |  | 36,542,052 (78.7%) | 29,043,385 (73.8%) |  | 9,990,104 (92.9%) | 7,946,567 (92.5%) |  | | 26,171 (96.7%) | | 25,996 (95.5%) | |  | | 6,998,683 (91.4%) | | 6,041,354 (92.8%) | |  | | 3,484 (81.5%) | | 3,081 (79.4%) | |  | | 67,767,926 (84.3%) | | | 54,915,999 (80.8%) |  |  |
| yes | 44,413,563 (22.3%) | 30,296,476 (15.7%) |  | 9,888,154 (21.3%) | 10,319,820 (26.2%) |  | 764,288 (7.1%) | 644,954 (7.5%) |  | | 902 (3.3%) | | 1,215 (4.5%) | |  | | 654,335 (8.6%) | | 471,022 (7.2%) | |  | | 789 (18.5%) | | 797 (20.6%) | |  | | 12,618,064 (15.7%) | | | 13,024,338 (19.2%) |  |  |
| **Diabetes** |  |  | **0.015** |  |  | **0.004** |  |  | **<0.001** | |  | |  | | **0.150** | |  | |  | | **0.700** | |  | |  | | **0.051** | |  | | |  | **<0.001** |  |
| no chronic | 175,351,368 (88.1%) | 173,617,482 (90.3%) |  | 36,358,202 (78.3%) | 29,961,507 (76.1%) |  | 8,016,066 (74.5%) | 6,884,466 (80.1%) |  | | 23,595 (87.2%) | | 23,575 (86.6%) | |  | | 6,346,957 (82.9%) | | 5,373,182 (82.5%) | |  | | 3,839 (89.9%) | | 3,386 (87.3%) | |  | | 68,456,708 (85.2%) | | | 56,208,985 (82.7%) |  |  |
| yes | 23,722,382 (11.9%) | 18,745,791 (9.7%) |  | 10,072,004 (21.7%) | 9,401,698 (23.9%) |  | 2,738,326 (25.5%) | 1,707,055 (19.9%) |  | | 3,478 (12.8%) | | 3,636 (13.4%) | |  | | 1,306,061 (17.1%) | | 1,139,194 (17.5%) | |  | | 433 (10.1%) | | 492 (12.7%) | |  | | 11,929,282 (14.8%) | | | 11,731,353 (17.3%) |  |  |
| **Arthritis** |  |  | **<0.001** |  |  | **<0.001** |  |  | **<0.001** | |  | |  | | **<0.001** | |  | |  | | **<0.001** | |  | |  | | **<0.001** | |  | | |  | **<0.001** |  |
| no chronic | 102,685,567 (51.6%) | 120,936,625 (62.9%) |  | 17,042,196 (36.7%) | 19,322,330 (49.1%) |  | 7,940,898 (73.8%) | 7,409,409 (86.2%) |  | | 22,645 (83.6%) | | 24,253 (89.1%) | |  | | 5,153,195 (67.3%) | | 5,947,737 (91.3%) | |  | | 2,467 (57.7%) | | 2,840 (73.2%) | |  | | 41,368,500 (51.5%) | | | 47,829,701 (70.4%) |  |  |
| yes | 96,388,184 (48.4%) | 71,426,648 (37.1%) |  | 29,388,010 (63.3%) | 20,040,875 (50.9%) |  | 2,813,494 (26.2%) | 1,182,112 (13.8%) |  | | 4,428 (16.4%) | | 2,958 (10.9%) | |  | | 2,499,823 (32.7%) | | 564,639 (8.7%) | |  | | 1,806 (42.3%) | | 1,038 (26.8%) | |  | | 39,017,489 (48.5%) | | | 20,110,636 (29.6%) |  |  |
| **Lung disease** |  |  | **<0.001** |  |  | **<0.001** |  |  | **0.002** | |  | |  | | **<0.001** | |  | |  | | **0.400** | |  | |  | | **0.900** | |  | | |  | **0.500** |  |
| no chronic | 174,103,226 (87.5%) | 159,377,983 (82.9%) |  | 41,528,135 (89.4%) | 35,917,160 (91.2%) |  | 9,771,277 (90.9%) | 8,064,292 (93.9%) |  | | 26,504 (97.9%) | | 26,449 (97.2%) | |  | | 7,446,923 (97.3%) | | 6,313,843 (97.0%) | |  | | 4,015 (94.0%) | | 3,640 (93.9%) | |  | | 72,522,085 (90.2%) | | | 61,065,857 (89.9%) |  |  |
| yes | 24,970,524 (12.5%) | 32,985,290 (17.1%) |  | 4,902,071 (10.6%) | 3,446,045 (8.8%) |  | 983,115 (9.1%) | 527,229 (6.1%) |  | | 568 (2.1%) | | 762 (2.8%) | |  | | 206,095 (2.7%) | | 198,533 (3.0%) | |  | | 258 (6.0%) | | 238 (6.1%) | |  | | 7,863,904 (9.8%) | | | 6,874,481 (10.1%) |  |  |
| **Stroke** |  |  | **<0.001** |  |  | **0.200** |  |  | **0.400** | |  | |  | | **<0.001** | |  | |  | | **0.200** | |  | |  | | **0.400** | |  | | |  | **0.110** |  |
| no chronic | 192,054,712 (96.5%) | 181,089,589 (94.1%) |  | 43,088,852 (92.8%) | 36,233,744 (92.0%) |  | 10,409,638 (96.8%) | 8,268,029 (96.2%) |  | | 26,629 (98.4%) | | 26,474 (97.3%) | |  | | 7,283,519 (95.2%) | | 6,145,419 (94.4%) | |  | | 4,098 (95.9%) | | 3,702 (95.5%) | |  | | 75,479,234 (93.9%) | | | 63,401,350 (93.3%) |  |  |
| yes | 7,019,038 (3.5%) | 11,273,684 (5.9%) |  | 3,341,354 (7.2%) | 3,129,461 (8.0%) |  | 344,754 (3.2%) | 323,492 (3.8%) |  | | 443 (1.6%) | | 737 (2.7%) | |  | | 369,499 (4.8%) | | 366,957 (5.6%) | |  | | 175 (4.1%) | | 176 (4.5%) | |  | | 4,906,755 (6.1%) | | | 4,538,987 (6.7%) |  |  |
| **Cancer** |  |  | **<0.001** |  |  | **0.600** |  |  | **0.018** | |  | |  | | **0.001** | |  | |  | | **0.009** | |  | |  | | **0.028** | |  | | |  | **0.004** |  |
| no chronic | 194,258,871 (97.6%) | 190,321,373 (98.9%) |  | 39,315,690 (84.7%) | 33,500,820 (85.1%) |  | 10,410,982 (96.8%) | 8,442,121 (98.3%) |  | | 26,849 (99.2%) | | 27,064 (99.5%) | |  | | 7,196,400 (94.0%) | | 6,221,017 (95.5%) | |  | | 3,755 (87.9%) | | 3,520 (90.8%) | |  | | 72,414,397 (90.1%) | | | 62,015,216 (91.3%) |  |  |
| yes | 4,814,879 (2.4%) | 2,041,900 (1.1%) |  | 7,114,516 (15.3%) | 5,862,385 (14.9%) |  | 343,410 (3.2%) | 149,400 (1.7%) |  | | 224 (0.8%) | | 148 (0.5%) | |  | | 456,618 (6.0%) | | 291,359 (4.5%) | |  | | 518 (12.1%) | | 358 (9.2%) | |  | | 7,971,592 (9.9%) | | | 5,925,121 (8.7%) |  |  |
| **Number of Conditions** |  |  | **<0.001** |  |  | **<0.001** |  |  | **<0.001** | |  | |  | | **<0.001** | |  | |  | | **<0.001** | |  | |  | | **<0.001** | |  | | |  | **<0.001** |  |
| no chronic | 68,432,541 (34.4%) | 79,570,990 (41.4%) |  | 10,537,872 (22.7%) | 11,075,867 (28.1%) |  | 5,226,551 (48.6%) | 5,186,315 (60.4%) |  | | 18,768 (69.3%) | | 19,441 (71.4%) | |  | | 3,794,099 (49.6%) | | 4,202,676 (64.5%) | |  | | 1,735 (40.6%) | | 1,896 (48.9%) | |  | | 29,197,080 (36.3%) | | | 30,180,372 (44.4%) |  |  |
| >=1 & <3 | 115,205,643 (57.9%) | 101,955,253 (53.0%) |  | 28,747,485 (61.9%) | 22,020,139 (55.9%) |  | 5,060,056 (47.1%) | 3,219,251 (37.5%) |  | | 8,116 (30.0%) | | 7,568 (27.8%) | |  | | 3,543,538 (46.3%) | | 2,208,415 (33.9%) | |  | | 2,234 (52.3%) | | 1,736 (44.8%) | |  | | 43,202,280 (53.7%) | | | 31,924,927 (47.0%) |  |  |
| >= 3 & <5 | 15,234,379 (7.7%) | 10,307,660 (5.4%) |  | 6,618,892 (14.3%) | 5,858,443 (14.9%) |  | 463,663 (4.3%) | 172,419 (2.0%) |  | | 187 (0.7%) | | 200 (0.7%) | |  | | 312,380 (4.1%) | | 101,285 (1.6%) | |  | | 297 (7.0%) | | 235 (6.0%) | |  | | 7,596,935 (9.5%) | | | 5,477,025 (8.1%) |  |  |
| >= 5 | 201,187 (0.1%) | 529,370 (0.3%) |  | 525,957 (1.1%) | 408,756 (1.0%) |  | 4,122 (0.0%) | 13,536 (0.2%) |  | | 1 (0.0%) | | 2 (0.0%) | |  | | 3,001 (0.0%) | | 0 (0.0%) | |  | | 7 (0.2%) | | 12 (0.3%) | |  | | 389,695 (0.5%) | | | 358,013 (0.5%) |  |  |
| ^1^n (%); Mean (SD) | | | |  | | |  |  | |  | |  | |  | |  | |  | |  | |  |  | | |  | |  |  | | |  |  |  |
| ^2^chi-squared test with Rao & Scott's second-order correction; Wilcoxon rank-sum test for complex survey samples | | | |  | | |  |  | |  | |  | |  | |  | |  | |  | |  |  | | |  | |  |  | | |  |  |  |
| * Data for India refers to year 2016/2017 and not 2014/2015. This was the closest year available to compare with other samples | | | | |  |  |  |  |  | |  | |  | |  | |  | |  | |  | |  | |  | |  | |  | | |  |  |  |
| ** only added in Wave 7 and thus not included in this study: Finland, Lithuania, Latvia, Slovakia, Romania, Bulgaria, Malta and Cyprus | | | | | | | | | |  | |  | |  | |  | |  | |  | |  | |  | |  | |  | |  | |  |  |  |

**SI References**

1. Saito Y, Robine JM, Crimmins EM. The methods and materials of health expectancy. Statistical journal of the IAOS. 2014;30(3):209–23.

2. Sullivan DF. A single index of mortality and morbidity. HSMHA health reports. 1971;86(4):347–54.

3. Horiuchi S, Wilmoth JR, Pletcher SD. A decomposition method based on a model of continuous change. Demography. 2008 Nov;45(4):785–801.

4. Riffe T. Package “DemoDecomp” Type Package Title Decompose Demographic Functions. 2018;

5. van Raalte AA, Nepomuceno MR. Decomposing Gaps in Healthy Life Expectancy. In: Jagger C., Crimmins E., Saito Y., De Carvalho Yokota R., Van Oyen H. RJ, editor. International Handbooks of Population. Springer, Cham; 2020. p. 107–22.

6. Nepomuceno MR, di Lego V, Turra CM. Gender disparities in health at older ages and their consequences for well-being in Latin America and the Caribbean. Vienna Yearb Popul Res. 2021 Dec 12;19.

7. Lee J, Phillips D, Wilkens J. Gateway to Global Aging Data: Resources for Cross-National Comparisons of Family, Social Environment, and Healthy Aging. J Gerontol B Psychol Sci Soc Sci. 2021 Jun 1;76(Suppl 1):S5.
