# Supplementary material for: Gender gaps in healthy life expectancy as indicators of inequality for disability and chronic disease: cross-sectional evidence from 24 countries, years 2014–2019
Source: BMJ Open. 2025 Nov 19;15(11):e096968. doi: 10.1136/bmjopen-2024-096968 (PMC12636968; doi:10.1136/bmjopen-2024-096968)
Supplement: Supplementary file 2 [file bmjopen-2024-096968.reviewer_comments.pdf]

PEER REVIEW HISTORY

BMJ Open publishes all reviews undertaken for accepted manuscripts. Reviewers are asked to complete a checklist review form and are provided with free text boxes to elaborate on their assessment. These free text comments are reproduced below.

ARTICLE DETAILS

Title (Provisional)

Gender Gaps in Healthy Life Expectancy as Indicators of Inequality for Disability and Chronic Disease: Evidence from 24 Countries, years 2014–2019

Authors

Di Lego, Vanessa; Nepomuceno , Marília R.; Turra, Cassio

VERSION 1 - REVIEW

**Reviewer**	**1**
**Name**	**Braun, Kathryn L.**
**Affiliation**	**University of Hawaii**
**Date**	**23-Jan-2025**
**COI**	**None**

I enjoyed reading this paper. Although I do not understand the mathematics (and other reviewer should check that), the research question was interesting and answered with solid data, illustrative figures, and good explanations. I was dismayed by the incomplete sentences in the abstract...is this the style of the journal?

**Reviewer**	**2**
**Name**	**Kelson, Zoe**
**Affiliation**	**University of Exeter, Mathematics**
**Date**	**17-Mar-2025**
**COI**	**None**

This study aims to assess whether gender gaps in healthy life expectancy are reliable markers of gender inequality in health.

Reviewer comments:

Can statistical outcomes please be reported in support of the Results in the Abstract?

"Data is from the Gateway to Global Aging, with harmonized versions of the Health and Retirement Study (HRS) sister studies from the Program on Global Aging, Health & Policy."

and

"We used data available for years 2014-2015 for HRS (United States of America), ELSA (England), KLoSA (South Korea), CHARLS (China), MHAS (Mexico), and Europe (SHARE), with the exception of India, where LASI was carried out between 2017-2019. An overview of the samples is displayed on Table 1."

Can the authors please confirm in the main article if the data used can be considered to be representative?

"All analyses are adjusted considering the weighted sample size and accounting for the complexity of each survey design." (Table 1 footnote)

and

"For more details on how the weights are constructed for each survey, refer to data documentation on https://g2aging.org/survey-overview ." [supplementary material]

Can the authors please provide more detail on how weighting was applied for each survey, perhaps in the supplementary material?

"However, a first analysis showed that in the sample of HRS and other countries more than 75% was missing for age variables. So, we did not use these variables in the analysis."

Can the authors please clarify how much missing data there was for each variable and sample?

How was missing data handled in the study?

"We first estimate disability-free life expectancy ( ) and chronic-disease-free life expectancy ( ) at age 60 using the Sullivan Method"

and

"we calculate the prevalence of individuals with limitations in disability and of at least one chronic disease for each country by 5-year age groups using the weighted proportions of women and men who reported these conditions in surveys and combine the estimated prevalence with country-specific life tables to compute healthy life expectancies.   is then defined as the number of years lived free of disability, while   is the number of years lived without chronic diseases. We then calculate the gender gap in   as Δ =   -   and the gender gap in   as Δ =   - n"

and

"Third, to decompose the gap into its health and mortality components, we apply the continuous change decomposition method, and split the gender differences in   and   at age 60 into mortality and disability/chronic effects"

The authors have followed a suitable approach, with detail provided in the supplementary material.

"Fig 1 shows that the prevalence of being diagnosed with at least one chronic disease is higher than that of a reported disability (Panel A). Most countries have a strong age gradient in health for both sexes (Panel B, see Figs S1-S8 in the SI for all countries and separately for each chronic disease and disability). Within countries, women experience a faster rate of increase in disability with age, resulting in greater health burden for women at younger ages relative to men"

Can statistical tests for significant differences between genders and over time within countries please be reported throughout the Results?

"Because the studies are longitudinal, participants in one wave can also be present in the other. We use the data as cross-sectional and focus on only one period."

Can the authors please clarify that there is no risk of participant duplication within the data (i.e. participant data can be treated as independent)?

"Based on cross-sectional data. • Uses a limited number of health variables and years in the analysis" [strengths and limitations of this study]

and

"It is important to acknowledge that this study has some limitations. Despite efforts to harmonize the variables, disease diagnosis is performed differently across countries. In some settings, the low prevalence of chronic diagnosed diseases may reflect the low quality of healthcare, such as in the case of India 40,55,56. Another relevant difference across countries is who can make the diagnosis. The HRS (United States of America) study, for example, specifically excludes diagnoses made by nurses/nurse practitioners, chiropractors, and dentists, while both CHARLS (China) and LASI (India) allow diagnoses by nurses, practitioners of traditional medicine, and other health care professionals. It is unclear whether these differences impact diagnoses for each gender in a similar way. However, the objective of this study is not to investigate the factors that determine health status in certain regions. Instead, the focus is on how differences between genders in terms of health expectancy incorporate various elements of health and mortality that raise doubts about its suitability as a measure of gender inequality in health. Also, our analyses do not use longitudinal data, only cross-sectional data for each country and for a limited period. Considering that health and mortality reflect transitions throughout the life course, it is possible that the assumption of a hypothetical cohort, inherent in cross-sectional data, may affect genders and countries in different ways, potentially biasing our comparisons. On the other hand, the use of cross-sectional data broadens the possibilities for comparison across a larger number of countries and has been used in the absence of longitudinal data."

A discussion on the study limitations has been provided by the authors.

Thanks for providing a copy of the STROBE checklist.

**Reviewer**	**3**
**Name**	**Roxo, Luis**
**Affiliation**	**Universidade Nova de Lisboa**
**Date**	**02-May-2025**
**COI**	**None**

The authors want to understand whether gender gaps in healthy life expectancy are reliable markers of gender inequality in health and they present evidence that shows that a similar gap in healthy life expectancy can be associated with different factors for different countries.

The paper is well written, it is interesting, have sufficient support from tables, figures and supplementary files. One can see that is was carefully written, and that is a very positive point.

Yet, I am not entirely satisfied and I believe that authors can make additional revisions to account for the following issues:

- The methods could be clearer. They are not easy to understand for those unfamiliar with this kind of methods. The decomposition can be easily explained using examples.

- Disability vs chronic conditions. You show that healthy life expectancy does not show covered different patterns of chronic diseases. That is relevant. But does it really show that healthy life expectancy is not appropriate? In the end, maybe the results show that in some countries, having chronic diseases does not impact disability. Maybe this is about objective vs subjective assessment of health. "Objective" measures (diagnoses) are not necessarily the best to assess health disparities. People can have chronic diseases and feel good about themselves, live without disability, etc.

- It seems that all data is presented as a comparison and I think that the absolute values could be important to interpret the results. The absolute values of healthy life expectancy can lead to different interpretations about the gender gap.

- Can you give more suggestions about which appropriate measures would be more suitable as reliable markers of gender inequality in health?

- Why age 60? Maybe at 60, chronic diseases have not translated into disability yet (?). What are the implications for your results?

I would like to read a comment for these comments. Do not change the manuscript if they do not make sense to you, but please explain the rationale to me. Also, some issues could be stated in the discussion/ limitations.

I made some comments on the pdf file.

**** The reviewer provided a marked copy with additional comments. Please contact the publisher for full details. ****

VERSION 1 - AUTHOR RESPONSE

Reviewer: 1

Prof. Kathryn L. Braun,

University of Hawaii

Comments to the Author:

Comment 1: I enjoyed reading this paper. Although I do not understand the mathematics (and other reviewer should check that), the research question was interesting and answered with solid data, illustrative figures, and good explanations. I was dismayed by the incomplete sentences in the abstract...is this the style of the journal?

Response:

Thanks for your remark; we are glad that the paper was interesting for you to read. We have corrected the incomplete sentences in the abstract. It is not the style of the journal but a formatting issue we faced when rendering the files for submission. Thanks for spotting this problem, as it allowed us to improve the abstract. Please, check the new version in the revised file.

Reviewer: 2

Prof. Zoe Kelson,

University of Exeter

Comments to the

Author:

Comment 1: This study aims to assess whether gender gaps in healthy life expectancy are reliable markers of gender inequality in health.

Can statistical outcomes please be reported in support of the Results in the Abstract?

Response: Yes, thanks for your request. We have rewritten the Abstract to include the statistical outcomes in support of the Results. Please refer to the tracked changes version, where we add the following:

"Results

Gender gaps in disability-free life expectancy ranged from -0.37 years (Portugal) to almost five years (South Korea), with most European countries showing female advantages of 3.0–3.5 years, while minimal gaps were observed in China, Mexico and India (0.4-0.9 years). Decomposition revealed striking inconsistencies between total gaps and underlying components—South Korea's 4.9-year gap reflected a survival advantage outweighing disability disadvantage by 13-fold, while Portugal's -0.37-year gap masked opposing contributions (mortality: +2.3; disability: -2.7). Chronic disease-free life expectancy showed female disadvantage in most countries, especially Portugal (-2.3), Korea (-1.6), and Mexico (- 1.9)".

Comment 2: "Data is from the Gateway to Global Aging, with harmonized versions of the Health and Retirement Study (HRS) sister studies from the Program on Global Aging, Health & Policy."

and

"We used data available for years 2014-2015 for HRS (United States of America), ELSA (England), KLoSA (South Korea), CHARLS (China), MHAS (Mexico), and Europe (SHARE), with the exception of India, where LASI was carried out between 2017-2019. An overview of the samples is displayed on Table 1."

Can the authors please confirm in the main article if the data used can be considered to be representative?

Response: Thank you for your comment. Yes, The HRS family surveys draw a nationally representative sample of the country's older population. They can be used as both longitudinal and cross-sectional surveys to reinterview the same individuals on an approximately biennial basis. We have added this information on the main manuscript in the Methods section, right after the Data and health variables subsection, starting on page 4*: "Data is from the Gateway to Global Aging, with harmonized versions of the Health and Retirement Study (HRS) sister studies [30] from the Program on Global Aging, Health & Policy. The HRS family surveys are designed to represent a nationally representative sample of the older adult population (ages 50 and above). "*

Comment 3: "All analyses are adjusted considering the weighted sample size and accounting for the complexity of each survey design." (Table 1 footnote)

and

"For more details on how the weights are constructed for each survey, refer to data documentation on https://g2aging.org/survey-overview ." [supplementary material]

Can the authors please provide more detail on how weighting was applied for each survey, perhaps in the supplementary material?

Response:

Thank you for your question. We have added more details in the Supplementary Material regarding the construction of weights and how we used them. Overall, the studies employed a post-stratification method to create these weights. We used the cross-sectional individual respondent-level weights provided for all surveys. Since each survey has its own design complexities, we included these details immediately following each data description. Please refer to the tracked changes version of the Supplementary Material under the heading "Country-Specific Details" for modifications we've made. We have added specific information for each country, as there are numerous surveys, and while they follow the Health and Retirement Study (HRS) guidelines, they remain country-specific. For instance, the U.S. oversamples Black respondents, while in India, districts are clustered. These unique aspects necessitate different approaches to weight construction.

Additionally, we included the following paragraph in the Methods section, specifically under the "Data and Health Variables" subsection in the Main Manuscript, to provide further clarity: *"HRS-sister studies utilize complex survey sample designs based on multi-stage probability samples and sometimes stratified cluster sampling. For more information on the specific sample designs and the construction of sample weights for each survey, please refer to the Supplementary Material under Country-Specific Details. We have used the cross-sectional individual-level respondent weights provided for all harmonized surveys."*

More information regarding weight construction and sampling design can be found on the Gateway to Global Aging website.

Comment 4: "However, a first analysis showed that in the sample of HRS and other countries more than 75% was missing for age variables. So, we did not use these variables in the analysis."

Can the authors please clarify how much missing data there was for each variable and sample? How was missing data handled in the study?

Response: Thanks for your careful reading of our paper. To clarify this point, we explicitly mention in the Supplementary Material, page 4, that this refers to variables that **were not used** in the analysis throughout the sample construction, specifically referring to variables of age at onset or age at diagnosis. We mentioned this because at first we wanted to include not only the age at prevalence of a condition, but also the age at diagnosis, to try to test differences in disease onset. However, these variables are not reliable since they also depend on respondents' reported age at diagnosis, even if confirmed by a doctor. We believe this information can be valuable for other researchers who seek to work with these data, which is why we included it in a robustness check, although we did not use it. Therefore, in the end, we investigated only the prevalence by age, which is more robust and was also the focus of our study.

For all the other health variables of interest, we had very few missing cases, thus our strategy was to drop the observations from the analyses. Importantly, there was no difference in the pattern of missing cases between women and men, which is crucial for our study. In response to the reviewer's request, we have included Table S1 in the Supplementary File, which includes the missing cases for every survey and variable used in the analysis, along with some notes specifically related to the survey.

Comment 5: "We first estimate disability-free life expectancy ( ) and chronic-disease-free life expectancy ( ) at age 60 using the Sullivan Method"

and

" we calculate the prevalence of individuals with limitations in disability and of at least one chronic disease for each country by 5-year age groups using the weighted proportions of women and men who reported these conditions in surveys and combine the estimated prevalence with country-specific life tables to compute healthy life expectancies. is then defined as the number of years lived free of disability, while is the number of years lived without chronic diseases. We then calculate the gender gap in as Δ = - and the gender gap in as Δ = - n" and

"Third, to decompose the gap into its health and mortality components, we apply the continuous change decomposition method, and split the gender differences in and at age 60 into mortality and disability/chronic effects"

The authors have followed a suitable approach, with detail provided in the supplementary material.

Response: Thank you for your comment.

Comment 6: "Fig 1 shows that the prevalence of being diagnosed with at least one chronic disease is higher than that of a reported disability (Panel A). Most countries have a strong age gradient in health for both sexes (Panel B, see Figs S1-S8 in the SI for all countries and separately for each chronic disease and disability). Within countries, women experience a faster rate of increase in disability with age, resulting in greater health burden for women at younger ages relative to men"

Can statistical tests for significant differences between genders and over time within countries please be reported throughout the Results?

Response: We appreciate your request because it helped us clarify our time unit of analysis**.** First, you can find in Table S6 in the Supporting Material a table with all the statistical tests for all surveys and descriptions for all health measures by gender. We do not have the results over time because

our work does not focus on trends over time; we look only into one point in time for all surveys. This was clarified in the Main Text (see page 6 immediately following the Methods section on Data and health variables), where we state:

"We selected the periods for which the greatest number of comparable surveys from various countries were available, taking into account all relevant health indicators. We focus on only one period; thus, we use the data as cross-sectional, accounting for respondents' health during a single period."

This is further discussed in Table 1's footnote. We have added in the Results section in parentheses all the tests whenever relevant.

Comment 7: "Because the studies are longitudinal, participants in one wave can also be present in the other. We use the data as cross-sectional and focus on only one period."

Can the authors please clarify that there is no risk of participant duplication within the data (i.e. participant data can be treated as independent)?

Response: Thank you for this important clarification. We can confirm there is no risk of participant duplication within our data, and all participant observations can be treated as independent for statistical analysis.

We use only one wave/period from each survey in our analysis, meaning each participant appears exactly once in our dataset. This eliminates any possibility of repeated observations from the same individual across different time points.

While the HRS-sister surveys are designed as longitudinal studies, we implemented a cross-sectional approach for several key methodological reasons. First, as we mentioned before, we selected periods with the largest number of comparable surveys across countries while ensuring all health indicators of interest were available and properly harmonized. Second, some surveys, such as LASI from India, had only one available wave, and harmonized health variables were not consistently available across all countries for all time periods. As this paper tests the reliability of gender gaps across diverse cultural contexts, maximizing the number of countries with comparable, well-harmonized data took priority over longitudinal analysis within countries.
We have clarified this approach in our manuscript through two key improvements. We corrected the imprecise footnote in Table 1, as noted in comment 6 above, and added clarifying language on page 6 following the Methods subsection, where we now state: *"We selected the periods for which the greatest number of comparable surveys from various countries were available, taking into account all relevant health indicators. We focus on only one period; thus, we use the data as cross-sectional, accounting for respondents' health during a single period."*

This cross-sectional approach ensures statistical independence of all observations while enabling the robust cross-national comparisons essential to our research objectives. The methodological trade-off between potential longitudinal analysis and cross-country comparability was necessary to address our primary research question about the reliability of gender health gaps across diverse cultural contexts.

Comment 8: "Based on cross-sectional data. • Uses a limited number of health variables and years in the analysis" [strengths and limitations of this study]

and

"It is important to acknowledge that this study has some limitations. Despite efforts to harmonize the variables, disease diagnosis is performed differently across countries. In some settings, the low prevalence of chronic diagnosed diseases may reflect the low quality of healthcare, such as in the

case of India 40,55,56. Another relevant difference across countries is who can make the diagnosis. The HRS (United States of America) study, for example, specifically excludes diagnoses made by nurses/nurse practitioners, chiropractors, and dentists, while both CHARLS (China) and LASI (India) allow diagnoses by nurses, practitioners of traditional medicine, and other health care professionals. It is unclear whether these differences impact diagnoses for each gender in a similar way. However, the objective of this study is not to investigate the factors that determine health status in certain regions. Instead, the focus is on how differences between genders in terms of health expectancy incorporate various elements of health and mortality that raise doubts about its suitability as a measure of gender inequality in health. Also, our analyses do not use longitudinal data, only cross-sectional data for each country and for a limited period. Considering that health and mortality reflect transitions throughout the life course, it is possible that the assumption of a hypothetical cohort, inherent in cross-sectional data, may affect genders and countries in different ways, potentially biasing our comparisons. On the other hand, the use of cross-sectional data broadens the possibilities for comparison across a larger number of countries and has been used in the absence of longitudinal data."

A discussion on the study limitations has been provided by the authors.

Comment: Thanks for providing a copy of the STROBE checklist.

Reviewer: 3

Dr. Luis Roxo, Universidade

Nova de Lisboa Comments to

the Author:

The authors want to understand whether gender gaps in healthy life expectancy are reliable markers of gender inequality in health and they present evidence that shows that a similar gap in healthy life expectancy can be associated with different factors for different countries.

The paper is well written, it is interesting, have sufficient support from tables, figures and supplementary files. One can see that is was carefully written, and that is a very positive point. Yet, I am not entirely satisfied and I believe that authors can make additional revisions to account for the following issues:

Comment 1: The methods could be clearer. They are not easy to understand for those unfamiliar with this kind of methods. The decomposition can be easily explained using examples.

Response: Thank you very much for your comment. We have rewritten the method's subsection "3. Decomposing the gender gap" of the Supplementary Material to clarify the method and make it more straightforward for readers unfamiliar with it. We also followed your suggestion and included an example. Please, see the revised version below, which can also be found in the supplementary material in tracked changes.

"We used a decomposition method to split the gender differences in Δ_xDFLE^ and Δ_xCFLE^ at ages 60 years and over into mortality and disability/chronic effects by five-year age groups. Decomposition methods are widely used tools to explain gaps in aggregate indices, such as life expectancies and healthy life expectancies. The goal of decomposition is to attribute gaps in aggregate indices to the contributions of underlying factors. For example, imagine two populations with different life expectancies. To understand why one population lives longer than the other, it is important to know which age groups contribute most to this difference. Decomposition methods allow us to break down the overall gap and determine, for example, how much is due to higher

infant mortality, how much stems from higher old-age mortality, and so on. In other words, instead of simply knowing that a gap exists, decomposition methods help us understand where the difference originates.

In this study, we apply the Continuous Change Decomposition Method 3 . By using this method, we assume that changes in mortality and health occur gradually between female and male populations 3–5. As van Raalte and Nepomuceno (2020) explain: "If we could split the time or space interval into many, many smaller intervals, then we could estimate the changes needed by the covariates (for instance age-specific mortality) to change the aggregate function (for instance life expectancy) from one population to the next". In this study, each tiny change in age-specific mortality and health measures in the healthy life expectancy can be approximated by a linear combination of n partial derivatives of the function to calculate healthy life expectancy with respect to its covariates: age-specific mortality and health measures. These can then be aggregated, using numerical integration to give the total contribution of the covariates to the change in the aggregate measure. This method is very flexible, and can be used for decomposing gaps in different aggregate measures. For more details, refer to 5. Previous research has employed this methodology to break down gender gaps in disability for Latin American and Caribbean (LAC) countries 6."

Additionally, we have added the example and how to interpret the decomposition in the main manuscript, on page 6, under the methods subsection "**Estimating and decomposing gender gaps in healthy life expectancy" :**

"Third, we decompose the gap into its health and mortality components. Decomposition methods are widely used tools to explain gaps in aggregate indices, such as life expectancies and healthy life expectancies. The goal of decomposition is to attribute gaps in aggregate indices to the contribution of underlying factors. For example, imagine two populations with different life expectancies. To understand why one population lives longer than the other, it is important to know which age groups contribute explaining this difference. Decomposition methods allow us to break down the overall gap and determine, for example, how much is due to higher infant mortality or how much stems from higher old-age mortality. In other words, decomposition methods help us understand where gaps comes from. In this paper, we apply the continuous change decomposition method [37–39], and split the gender differences in and at age 60 into mortality and disability/chronic effects (See SM section on Methods for more details on the Sullivan method and the decomposition approach). This allows us to estimate the contribution of health and mortality to explaining the gap between women and men.

The sum of these two components corresponds to the total gender gap. When the total gender gap is positive, it indicates that women have higher healthy life expectancy than men, which is known as women's advantage in healthy life

expectancy. In such cases, when both components of mortality and health are positive, they both increase the gender gap. On the other hand, when one component is positive, and the other is negative, they result in a narrower gap."

Comment 2: Disability vs chronic conditions. You show that healthy life expectancy does not show covered different patterns of chronic diseases. That is relevant. But does it really show that healthy life expectancy is not appropriate? In the end, maybe the results show that in some countries, having chronic diseases does not impact disability. Maybe this is about objective vs subjective assessment of health. "Objective" measures (diagnoses) are not necessarily the best to assess health disparities. People can have chronic diseases and feel good about themselves, live without disability, etc.

Response: Thank you for your careful reading and raising this discussion on the topic.
First and foremost, our goal is not to demonstrate that healthy life expectancy is inappropriate to show different patterns of disease or to measure health. Our goal is to specifically indicate that gender gaps in healthy life expectancy must be interpreted with caution since they may not yield accurate measures of inequality in health between women and men. What we often see in the

literature are gender gaps being used as indicators of inequality and as a starting point to discuss public health policy or monitor countries. In this paper, we would like to raise awareness that, unlike gaps in total life expectancy, gaps in healthy life expectancy between women and men can mask important health differences, with misleading results.
We have added clarifying sentences in the Introduction (see page 4) stating that *"while healthy life expectancy remains a valuable health measure, our focus is on examining whether gender gaps in these measures appropriately capture inequality between women and men,'* and in the Results (see pages 12-13) emphasizing that *'the contrasting patterns between disability-free and chronic disease-free life expectancy demonstrate how relying only on gap indicators can mask important underlying health differences".*

Second, you are right about the objective vs. subjective assessment of health. People can indeed "objectively" have chronic diseases and "subjectively" feel good about themselves, especially if their chronic condition is being controlled for with treatment. It is also true that depending on environmental (good street pavement, safe neighborhoods) and infrastructural conditions (having access to devices that mitigate one's disability, such as a walking cane, hearing device, or getting help from another person), a person may have a disability "objectively" but "subjectively" state that they feel good and not impaired in their activities of daily living. However, our goal in this section was not to specifically compare disability with chronic conditions across individuals; it was to demonstrate that the issue with misinterpretation holds regardless of whether one measures health defined as a state of limitation or chronic disease. As it is widely acknowledged in health research, the first challenge in measuring health is defining health in the first place. It is common knowledge that, depending on the health domain, different results may be reached. In the particular case of gender differences, research has focused on disability and chronic conditions, as they are the two groups of health dimensions that most importantly impact women's and men's health. Hence, we demonstrate that regardless of which health domain we investigate, or how we define health, the issue with misinterpreting gender gaps in health expectancy as accurate measures of inequality still holds. To make this idea clearer we have further the following sentence on the first paragraph of the Discussion, page 13: *"Our findings show that the issue with misinterpreting gender gaps as accurate inequality measures holds regardless of which health domain is investigated or how health is defined."*

Comment 3: It seems that all data is presented as a comparison and I think that the absolute values could be important to interpret the results. The absolute values of healthy life expectancy can lead to different interpretations about the gender gap.

Response: Thank you for your comment. Indeed, we have focused on presenting our results in terms of gender gaps in healthy life expectancy since the core issue we are discussing is the use of gender gaps themselves as metrics of inequality in health. In response to your request, we have added Tables S2 and S3 in the Supplementary Files with all gender gap values both in total life expectancy and healthy life expectancy, with confidence intervals for each health dimension. You will see that conclusions do not change, since what reveals the issues with the disparities is the decomposition.
Comment 4: Can you give more suggestions about which appropriate measures would be more suitable as reliable markers of gender inequality in health?

Response: Thank you for your request. We have added some alternative/complementary suggestions in the Discussion section (see page 14). In principle, HLE gender gaps can be informative markers of inequality—but only when disaggregated by age, type of disability, and social context. Other indicators that could more accurately reflect important gender disparities are: Severity-adjusted life expectancy distinguishing mild vs. severe disabilities; Cause-specific mortality ratios by gender across different health conditions; Gender gaps in healthcare utilization and access; Preventable mortality ratios by gender; Health system responsiveness measures by gender.

Comment 5: Why age 60? Maybe at 60, chronic diseases have not translated into disability yet (?). What are the implications for your results?

Response: Thanks one more time for your valuable comment which allowed us to clarify this point further. First, we focus not only on age 60, **but on ages 60 and above,** so here is included the group of older individuals that experience a higher prevalence of disability and chronic conditions, as shown in Figure 1. Recalling that, as in response to your second comment, our goal was not to evaluate how chronic disease translates into disability per se, but how, for these two critical health domains, we must be equally careful when interpreting gaps in healthy life expectancy.
Second, most reports that use gender gaps in healthy life expectancy to evaluate gender inequalities and implement policy action for health equity as mentioned in the Introduction Section (The Global Gender Gap reported by the World Economic Forum with data from the World Health Organization, the WHO European Health Equity Status Report initiative (HESRi), and the Gender Equality Index are examples of such indicators) use either gender gaps at birth or at ages 60 and above. Since the majority of health surveys, like the ones we use, only include older individuals, we have focused our study on that age group. Lastly, it is noteworthy to mention that it has been documented that the greatest gender disparities in health for disability and chronic conditions become more pronounced starting from age 60. To clarify our choice and explain why we present the results for age 60, we have included the following sentence in the Methods Section:
"We focus on healthy life expectancy at age 60 because this age marks the onset of more pronounced gender disparities in health outcomes, follows the methodology used by major international reports that monitor gender health inequalities, and corresponds to the age range captured by the health surveys in our analysis."

I would like to read a comment for these comments. Do not change the manuscript if they do not make sense to you, but please explain the rationale to me. Also, some issues could be stated in the discussion/ limitations.

I made some comments on the pdf file.

VERSION 2 - REVIEW

**Reviewer**	**3**
**Name**	**Roxo, Luis**
**Affiliation**	**Universidade Nova de Lisboa**
**Date**	**23-Sep-2025**
**COI**	

Thank you for addressing my main comments. I have no further comments on that regard.

I have made small comments on the pdf file that were not addressed. Can you please provide a reply for them, and make the necessary changes?

INTRODUCTION

"69 Researchers often use gender gaps in healthy life expectancy to evaluate health disparities

70 between women and men"

I think it would be good to state that gender differences in life expectancy are favorable to women, and gender gaps in healthy life expectancy are favorable to men. And why this happens (comparison between patterns of disease of men and women).

This appears later, but I believe that in the beginning there should be a reference to this "paradox", to help understand the first paragraphs.

"This is because the gap is due to both gender differences in health

79 prevalence and mortality rates by health status"

I do not understand this sentence. Is this how you compute gender gaps in healthy life expectancy? What is health prevalence? How do you calculate that?

"However, including

86 other health domains in indicators - as is the case with healthy life expectancy - is much more

87 complex"

Why is it so complex? The comparison seems similar to the one made for life expectancy. What seems different and complex to me is the calculation of healthy life expectancy.

"While population health and mortality are closely linked, there are substantial differences in how

89 these two factors affect men and women."

I get the general idea, but population health and mortality are not "factors" that "affect" men and women. There are differences in determinants, outcomes, years lived, causes of death, etc...

"These factors make it challenging to interpret gender gaps in health

93 as markers of inequality"

I do not think it is entirely clear what you want to say here. You would want to interpret gender gaps in health as markers of inequality? Why?

What do you mean by gender gaps in health? Healthy life expectancy? Or this higher morbidity?

What do you mean by markers of inequality? Societal gender inequality?

RESULTS

Is it the same information plotted in different ways in Panel A and Panel B?

"which could be due to limited

225 access to healthcare, as these diseases require diagnoses by a medical doctor. Previous

226 research indicates that underdiagnosis is common in India"

I would leave interpretations to the discussion section.

"Fig 2 shows the total gender gap in disability-free life expectancy ( ) by country and world

229 region"

For world region? Are you showing that? Or are you just presenting by region?

In Figure 2, South Korea is referred as "Korea". Please confirm what is the correct name of this country.

"due to women's remarkable survival advantage"

I would avoid using adjectives like this in the results. And why is it remarkable? I would say the goal is to decrese inequalities overall and improve the health status of everyone, not for one group to have "remarkable" outcomes compared to others...

"The contributions of both disability and mortality are remarkably high"

Contribution of disability is among the highest, but contribution from mortality is actually among the lowest in Europe.

VERSION 2 - AUTHOR RESPONSE

Reviewer: 3

Dr. Luis Roxo, Universidade Nova de Lisboa

Comments to the Author:

Thank you for addressing my main comments. I have no further comments on that regard.

I have made small comments on the pdf file that were not addressed. Can you please provide a reply for them, and make the necessary changes?

Remarks raised by each section of the paper:

INTRODUCTION

QUESTION 1: "Researchers often use gender gaps in healthy life expectancy to evaluate health disparities between women and men". I think it would be good to state that gender differences in life expectancy are favorable to women, and gender gaps in healthy life expectancy are favorable to men. And why this happens (comparison between patterns of disease of men and women). This appears later, but I believe that in the beginning there should be a reference to this "paradox", to help understand the first paragraphs.

RESPONSE: Thanks for your comment. You are right, we should define here already what we mean by gender gaps in healthy life expectancy and how they present differently for women and men. We have thus shifted the definition that comes later and start the INTRODUCTION like this:

It has been widely documented that women live longer than men, but spend more years in poorer physical, self-rated, and cognitive health at older ages [1–6]. They also experience higher morbidity from acute and chronic conditions, and more short-term disability [7–10]. This phenomenon is known in the literature as the male-female health-survival paradox, and researchers often use gender gaps in healthy

life expectancy to evaluate health disparities between women and men [11–15]*. When it comes to healthy life expectancy, gender gaps are calculated by comparing the number of years that women and men are expected to live in good health* [13,16,17]*. This comparison can be expressed as a ratio or an absolute difference. These measures are then used to make inferences about gender discrepancies in health and whether women and men have higher or lower inequality in terms of health and mortality in a population* [2,7,18,19]*. Policymakers also rely on such gender gaps to benchmark countries, monitor changes over time and age, and assess whether countries are closing or widening gender inequality in health* [20–22]*.*

QUESTION 2: "This is because the gap is due to both gender differences in health prevalence and mortality rates by health status". I do not understand this sentence. Is this how you compute gender gaps in healthy life expectancy? What is health prevalence? How do you calculate that?

RESPONSE: Thank you for your comment that allows us to further clarify this point. Yes, this is linked to how one computes gender gaps in healthy life expectancy. This is defined in the Methods section and further detailed in the Supplementary File. To make this point clear to the reader before reaching the Methods section, we have added in this same paragraph the following (page 4, starting line 136): *"This is due to the way that gaps in healthy life expectancy are estimated, incorporating both the proportion of women and men who are unhealthy at a specific age and their mortality rates. The absolute difference in years between their healthy life expectancy is then used as a gender gap in health and mortality. Gaps are often used to measure the difference between two quantities*[24]*. These measures are then used to make inferences about gender discrepancies in health* [2,7,18,19]*. Indeed, gaps can be robust ways to compare quantities like life expectancy, which depend solely on mortality – a single state that represents the very final stage of the decline of one's health. However, including other health domains in indicators - as is the case with healthy life expectancy – leads to much more complex interpretations* [25]*."*

QUESTION 3 "However, including other health domains in indicators - as is the case with healthy life expectancy - is much morecomplex". Why is it so complex? The comparison seems similar to the one made for life expectancy. What seems different and complex to me is the calculation of healthy life expectancy.

RESPONSE: Thank you for your comment. We meant here that contrary to life expectancy, on which only one uncontroversial health state is analysed (i.e., death), healthy life expectancy is much more complex, because we include health, a multidimensional indicator in itself. When we compare life expectancy, we are indeed only comparing the mortality experience of different subgroups calculated using life tables, in this case, for women and men. However, when we compare healthy life expectancy, we are mixing both health and mortality, since, as mentioned in the response above, healthy life expectancy incorporates both. Because women and men are differently impacted by mortality and health, we can generate inaccurate comparisons.

That is why this sentence comes right after the following: *"Indeed, gaps can be robust ways to compare quantities like life expectancy, which depend solely on mortality – a single state that represents the very final stage of the decline of one's health."*

However, the more important point here is the consequence/interpretation of using gaps in healthy life expectancy, so to make it clearer, we have adjusted this sentence slightly to (see line 145, page 4):

"However, including other health domains in indicators - as is the case with healthy life expectancy – leads to much more complex interpretations [25]*."*

QUESTION 4 "While population health and mortality are closely linked, there are substantial differences in how these two factors affect men and women." I get the general idea, but population health and mortality are not "factors" that "affect" men and women. There are differences in determinants, outcomes, years lived, causes of death, etc...

<u>RESPONSE:</u> Yes, you are right that our choice of words here does not best reflect what we mean. We have changed this sentence to (page 4 line 147):

"While population health and mortality are closely linked, they each exert different impacts on women and men."

QUESTION 5 "These factors make it challenging to interpret gender gaps in health as markers of inequality". I do not think it is entirely clear what you want to say here. You would want to interpret gender gaps in health as markers of inequality? Why?

<u>RESPONSE:</u> Thanks for your comment, since this is linked to our main argument for the paper and to what we present early on in the INTRODUCTION (see lines 117-124, where we state : *"Gaps in healthy life expectancy are also used to construct aggregate indicators of gender inequality. The Global Gender Gap reported by the World Economic Forum with data from the World Health Organization [20], the WHO European Health Equity Status Report initiative (HESRi), and the Gender Equality Index are examples of such indicators. They are often used to evaluate gender inequalities in health and mortality [21] and implement policy action for health equity [23].... However, it can be misleading to use gender gaps in healthy life expectancy as indicators of gender inequality in health".* It is not us who suggest to interpret gender gaps in health as markers of inequality, but the current research and also official reports that do so. The gaps, calculated as the absolute difference between healthy life expectancy, are used by various reports and scholars as an indicator of inequality, and we are precisely saying that one needs to be careful when adopting that strategy/perspective, since they do not necessarily measure inequality in health between women and men. The reason why gender gaps in healthy life expectancy do not measure inequality in the way that scholars and policy makers often believe they do is that they mix information from both health and mortality, each of which impacts women and men differently. We believe that by making the requested changes in QUESTIONS 1-3 it better connected to this sentence and main argument, making it explicit what we mean by inequality in this context. We have additionally slightly changed the sentence to reflect that we refer explicitly to healthy life expectancy in terms of inequality (see line 148 pages 4-5: *"Hence, it is challenging to interpret gender gaps in healthy life expectancy as markers of inequality, as many aspects can be overlooked")*

QUESTION 6 What do you mean by gender gaps in health? Healthy life expectancy? Or this higher morbidity?

<u>RESPONSE:</u> Thanks for your comment and sorry for our imprecision here. We mean how **differences in healthy life expectancy between** women and men have been measured traditionally in the field of demography and population health studies, where the gap in healthy life expectancy is measured by the absolute difference in years in healthy life expectancy between women and men. This indicator can be estimated using any health dimension of interest, ie., chronic- free disease life expectancy, active life expectancy, etc. We have focused on the two most commonly used health dimensions to demonstrate our point that gaps in healthy life expectancy are not accurate indicators of inequality, regardless of the health dimension one looks at. In order to make this clearer, we have added healthy life expectancy whenever we refer to this specifically.

QUESTION 7 What do you mean by markers of inequality? Societal gender inequality?

RESPONSE: No, I mean gender gaps in healthy life expectancy. Our response to your QUESTION 5 above has also made this clear. It has been common practice in the literature to use gender gaps in healthy life expectancy as important markers of inequality in health and mortality. If the gender gap in healthy life expectancy is wide, it is considered that we have higher inequality; if the gender gap is narrower, it is considered lower inequality. It is in that sense. The newly added sentences and remarks in response to QUESTIONS 1-5 have hopefully made this point clearer.

RESULTS

QUESTION 8 Is it the same information plotted in different ways in Panel A and Panel B?

RESPONSE: Essentially yes, but focusing on different aspects of the problem. While Panel A focuses on the prevalence (% of the total population that has a health condition), Panel B focuses on the age gradient in health for both sexes, by highlighting some countries and having the curve-line graph selecting a subset of countries. While Panel A is a heatmap that shows all countries and the magnitude by age, Panel B selects a few cases that are more discussed throughout the paper and highlights the pattern by age. The idea is that one complements the other, as mentioned in the notes in the caption of the Figure.

QUESTION 9 "which could be due to limited access to healthcare, as these diseases require diagnoses by a medical doctor. Previous research indicates that underdiagnosis is common in India". I would leave interpretations to the discussion section.

RESPONSE: Yes, you are right, and we actually cover this topic in the DISCUSSION. Thanks for your comment, we have dropped that sentence from the RESULTS section.

QUESTION 10 "Fig 2 shows the total gender gap in disability-free life expectancy (DFLE) by country and world region". For world region? Are you showing that? Or are you just presenting by region?

RESPONSE: Thanks for spotting this. Just presenting by region, we have droped the term world region.

QUESTION 11 In Figure 2, South Korea is referred as "Korea". Please confirm what is the correct name of this country.

RESPONSE: Thanks for your attentive read and spotting this. Yes, it is South Korea in the whole paper and Figure 2. We have adjusted accordingly and added a note in the Figure caption.

QUESTION 12 "due to women's remarkable survival advantage". I would avoid using adjectives like this in the results. And why is it remarkable? I would say the goal is to decrease inequalities overall and improve the health status of everyone, not for one group to have "remarkable" outcomes compared to others...

RESPONSE: Thank you. We used “remarkable” here in the sense of striking, because the differential is very high, but you are right that it has a strong normative tone, and that was not our intention. We have dropped the adjective from the sentence.

QUESTION 13 "The contributions of both disability and mortality are remarkably high". Contribution of disability is among the highest, but contribution from mortality is actually among the lowest in Europe.

RESPONSE: Thank you for your attentive reading. Yes, you are right, but here we mean not only in terms of absolute magnitude, but in the sense of total contribution to the gaps in healthy life expectancy, while stressing the case for Portugal in specific. Here it is clear that one person may have authorization but she sill had to shoe her the opic, That is why it follows the study in Portugal. In order to make this point clearer, we have excluded the word “remarkable”.
